# A novel allele of *ASY3* is associated with greater meiotic stability in autotetraploid *Arabidopsis lyrata*

**DOI:** 10.1371/journal.pgen.1008900

**Published:** 2020-07-15

**Authors:** Paul J. Seear, Martin G. France, Catherine L. Gregory, Darren Heavens, Roswitha Schmickl, Levi Yant, James D. Higgins

**Affiliations:** 1 Department of Genetics and Genome Biology, University of Leicester, Leicester, United Kingdom; 2 Earlham Institute, Norwich Research Park Innovation Centre, Norwich, United Kingdom; 3 Department of Botany, Faculty of Science, Charles University, Prague, Czech Republic; 4 Institute of Botany, The Czech Academy of Sciences, Průhonice, Czech Republic; 5 Future Food Beacon of Excellence and the School of Life Sciences, University of Nottingham, Nottingham, United Kingdom; INRA, FRANCE

## Abstract

In this study we performed a genotype-phenotype association analysis of meiotic stability in 10 autotetraploid *Arabidopsis lyrata* and *A*. *lyrata/A*. *arenosa* hybrid populations collected from the Wachau region and East Austrian Forealps. The aim was to determine the effect of eight meiosis genes under extreme selection upon adaptation to whole genome duplication. Individual plants were genotyped by high-throughput sequencing of the eight meiosis genes (*ASY1*, *ASY3*, *PDS5b*, *PRD3*, *REC8*, *SMC3*, *ZYP1a/b*) implicated in synaptonemal complex formation and phenotyped by assessing meiotic metaphase I chromosome configurations. Our results reveal that meiotic stability varied greatly (20–100%) between individual tetraploid plants and associated with segregation of a novel *ASYNAPSIS3* (*ASY3*) allele derived from *A*. *lyrata*. The *ASY3* allele that associates with meiotic stability possesses a putative in-frame tandem duplication (TD) of a serine-rich region upstream of the coiled-coil domain that appears to have arisen at sites of DNA microhomology. The frequency of multivalents observed in plants homozygous for the *ASY3 TD* haplotype was significantly lower than in plants heterozygous for *ASY3 TD/ND* (non-duplicated) haplotypes. The chiasma distribution was significantly altered in the stable plants compared to the unstable plants with a shift from proximal and interstitial to predominantly distal locations. The number of HEI10 foci at pachytene that mark class I crossovers was significantly reduced in a plant homozygous for *ASY3 TD* compared to a plant heterozygous for *ASY3 ND/TD*. Fifty-eight alleles of the 8 meiosis genes were identified from the 10 populations analysed, demonstrating dynamic population variability at these loci. Widespread chimerism between alleles originating from *A*. *lyrata/A*. *arenosa* and diploid/tetraploids indicates that this group of rapidly evolving genes may provide precise adaptive control over meiotic recombination in the tetraploids, the very process that gave rise to them.

## Author summary

Whole genome duplication can promote adaptability, but is a dramatic mutation usually resulting in meiotic catastrophe and genome instability. Here we focus on a case of coordinated stabilization of meiotic recombination in 10 autotetraploid *Arabidopsis lyrata* and *A*. *lyrata/A*. *arenosa* hybrid populations from the Wachau region and East Austrian Forealps. We fuse population genomic data with a genotype-phenotype association study, concentrating on the effects of eight meiosis genes (*ASY1*, *ASY3*, *PDS5b*, *PRD3*, *REC8*, *SMC3*, *ZYP1a* and *ZYP1b*) implicated in synaptonemal complex formation in the tetraploids under extreme selection. Our analysis demonstrates that segregation of a novel allele of the meiotic chromosome axis protein ASYNAPSIS3 is associated with male meiotic stability. Adaptive restabilisation of meiotic recombination appears to be achieved by a reduction in the number of crossovers as well as a shift in their positioning towards the chromosome ends. Of the eight genes, 58 alleles were identified, indicating dynamic population variability at these loci under extreme selection. In addition, widespread allelic chimerism between alleles originating from *A*. *lyrata/A*. *arenosa* and diploid/tetraploids indicates that this group of rapidly evolving genes may provide precise adaptive control over meiotic recombination in the tetraploids, the very process that gave rise to them.

## Introduction

Whole genome duplication (WGD) occurs in all eukaryotic kingdoms, and is associated with adaptability, speciation and evolvability [1, 2]. At the same time, it is also one of the most dramatic mutations observed, usually resulting in catastrophic problems during meiosis, when ensuring stable chromosome segregation and genome integrity is paramount [3]. Because efficient meiosis is required for the formation of euploid gametes during sexual reproduction, selection acts strongly on standing variation from the progenitor diploids in newly arisen polyploids.

In allopolyploids (formed by both genome duplication and interspecies hybridization), loci required for correct chromosome pairing and recombination have been identified in wheat [4], oil seed rape [5, 6] and *Arabidopsis suecica* [7]. However, in autopolyploids (which form within-species, without hybridization), there has been little functional confirmation of any gene controlling correct chromosome pairing, synapsis and crossing over (CO), although clear signatures of extreme selection have been detected in eight meiosis genes associated with the synaptonemal complex (SC) (*ASY1*, *ASY3*, *PDS5b*, *PRD3*, *REC8*, *SMC3*, *ZYP1a*, *ZYP1b*) in the young autotetraploid *Arabidopsis arenosa* [8].

The SC is a tripartite protein structure consisting of two lateral elements and a central element, specific to meiotic prophase I that is required for normal levels of COs in the majority of sexually reproducing eukaryotes [9]. In Arabidopsis, the chromosome axes (which come to form the SC lateral elements) consist of a scaffold of cohesin proteins (SMC1, SMC3, PDS5, REC8 and SCC3)[10–14] that organise sister chromatids into a loop/base conformation [15]. PRD3, the budding yeast MER2 homolog, is required for double-strand break (DSB) formation and is not an SC protein *per se* but may juxtapose the potential DSB site with the chromosome axis to promote inter-homolog recombination [16, 17]. In *Sordaria*, MER2 also transfers and releases recombination complexes to and from the SC central region [16]. The meiosis specific proteins ASY1, ASY3 and ASY4 load onto the chromosome axis defined by the cohesin scaffold, to promote inter-homolog recombination [18–20]. ASY1 and ASY3 are the functional homologs of budding yeast HOP1 and RED1 and, HORMAD1/2 and SCP2 in mammals, respectively, that facilitate correct chromosome pairing and synapsis, required for wild-type COs [19, 21, 22]. In Arabidopsis, synapsis is initiated by installation of the transverse filament proteins ZYP1a and ZYP1b between homologous chromosomes, thus ensuring appropriate levels of COs [23].

*Arabidopsis lyrata* and *A*. *arenosa* represent powerful models for investigating adaptation to autopolyploidy, particularly their populations from the eastern Austrian Forealps where interspecific hybridization and introgression is frequent [8] [24–26]. Extensive demographic analysis and coalescent modelling demonstrated that each of these species first underwent WGD and then later hybridised intraspecifically, only between their autotetraploid cytotypes [27]. Independent studies of the ploidy specific hybridisation barrier [28] functionally confirm that these species only hybridise following WGD, after each is established in its autopolyploid form. As a consequence, these populations represent ‘natural mapping experiments’ that can be studied to understand the relative contributions of the suite of alleles known to exhibit strong signatures of selection. These eight meiosis loci displaying highly differentiated alleles in *A*. *arenosa* were also reported in *A*. *lyrata* autotetraploids, along with signatures of extensive bidirectional gene flow [25]. At meiotic metaphase I in *A*. *arenosa*, chiasma frequency was reduced in autotetraploids carrying the derived alleles compared to the diploids, indicating an ongoing adaptive consequence of their evolution [8]. Recently *ASY1* and *ASY3* have been shown to have an effect in stabilising autotetraploid *A*. *arenosa* [29], but thus far, no formal confirmation of an association between meiotic stabilisation has been attributed to these evolved alleles in tetraploid *A*. *lyrata*.

In this study we fuse genomic, genetic and cytological approaches to investigate the effects of rapidly evolving adaptive haplotypes in these meiosis genes under strong selection. We measure the consequences of alternative haplotypes at these loci in autotetraploid *A*. *lyrata*, *A*. *arenosa*, and natural introgressants of these species across a hybrid zone. Our analysis reveals an association between a novel *ASY3* haplotype and meiotic chromosome stability.

## Results

### A metaphase I analysis to determine meiotic stability in *A*. *lyrata*/*A*. *arenosa* populations

**Fig 1:**
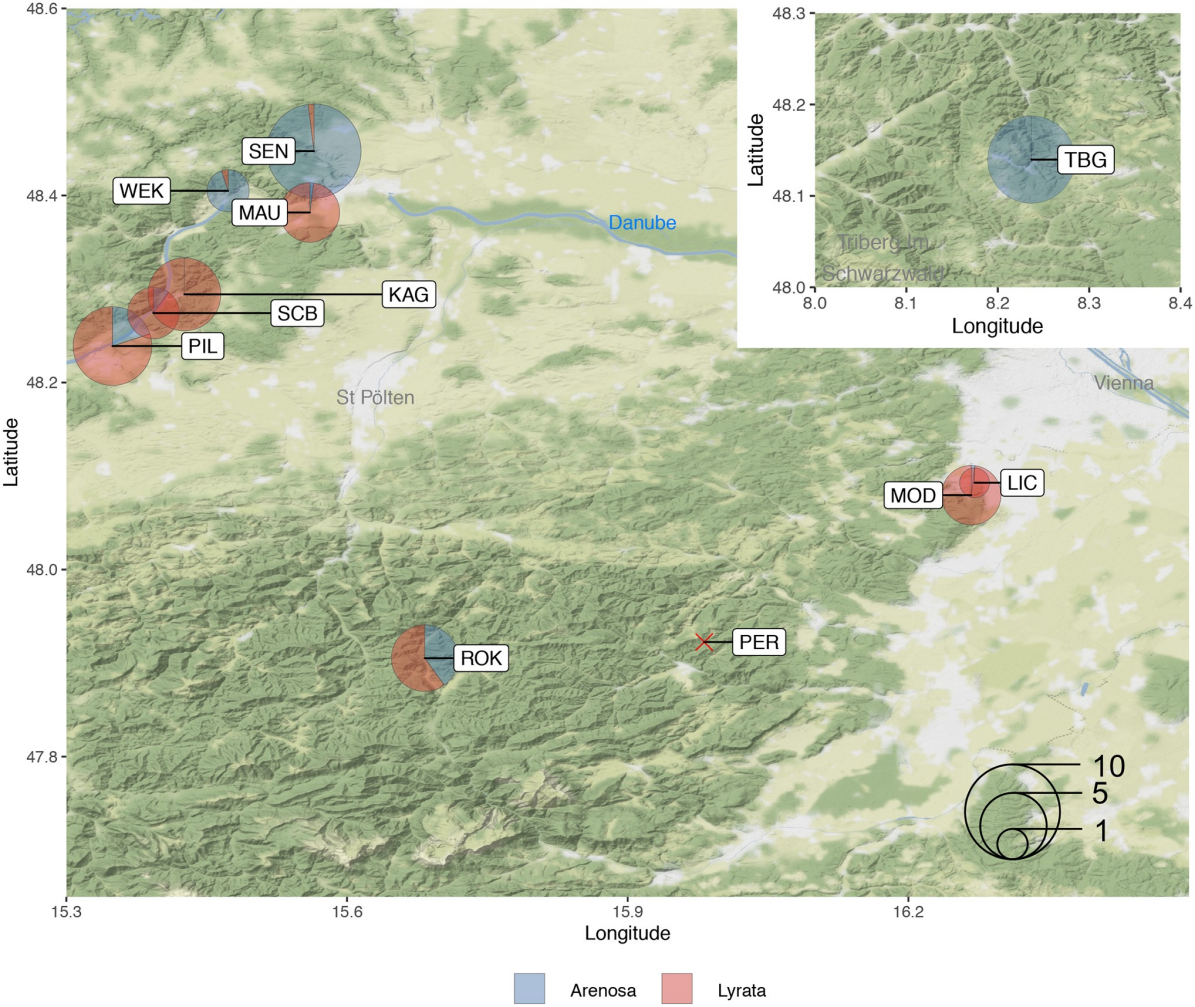
Map of maternal seed lines collection sites from the eastern Austrian fore Alps and Wachau valley. Collection sites are indicated as circles for tetraploid plants and an X for diploids. Pie chart circle size represents the numbers of plants sampled and analysed from each site and the proportion indicates the relative amounts of admixture between *A*. *lyrata* (red) and *A*. *arenosa* (blue) populations determined in [25].

**Fig 2:**
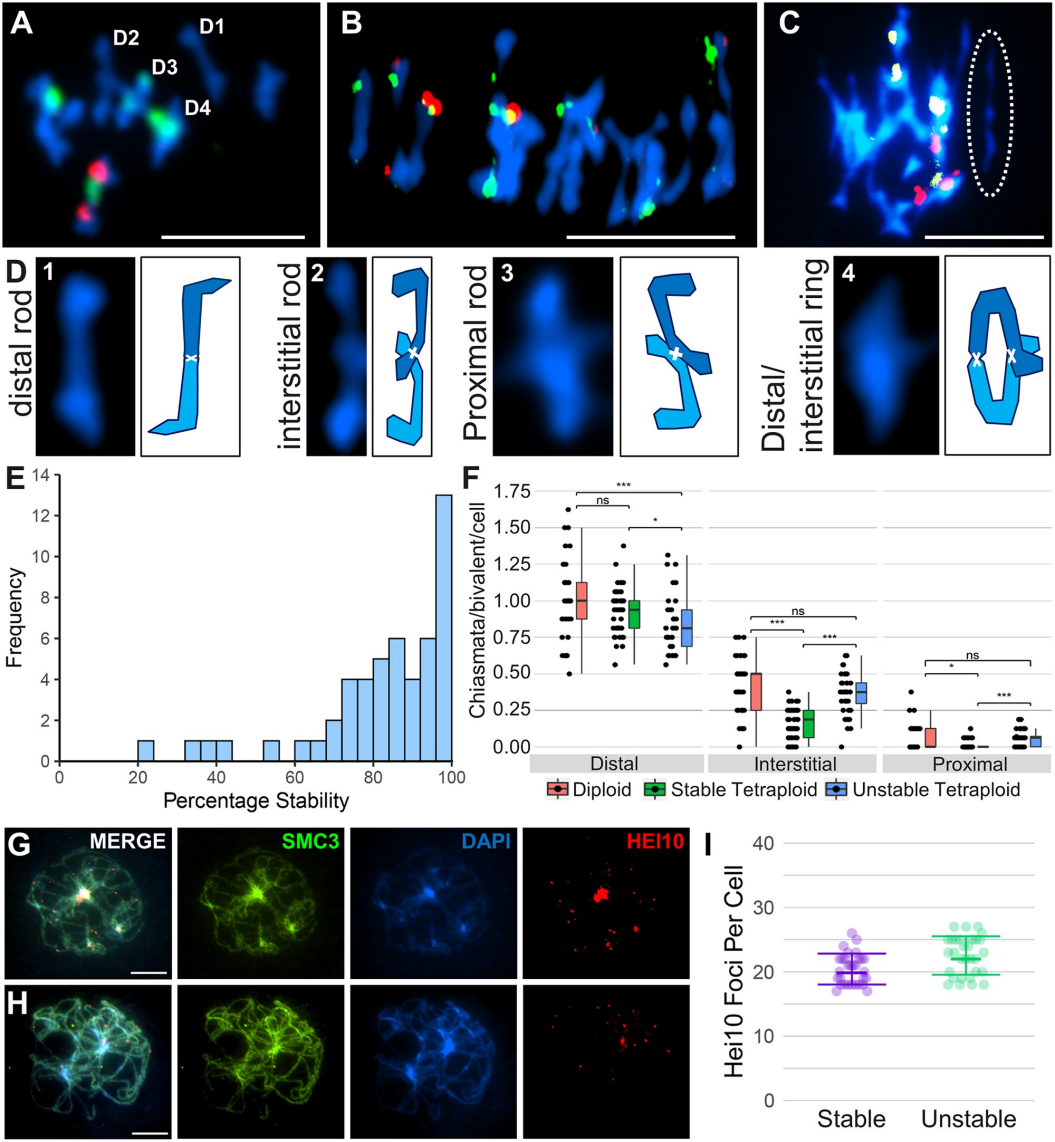
Cytological analysis of meiotic metaphase I stability, chiasma frequency and distribution. (A) Diploid *A*. *lyrata* bivalents stained with DAPI (blue) 5*S* rDNA (red) and 45*S* rDNA (green). (B) Stable autotetraploid meiotic metaphase I, stained with DAPI (blue) 5*S* rDNA (red) and 45*S* rDNA (green). (C) Unstable autotetraploid meiotic metaphase I, stained with DAPI (blue) 5*S* rDNA (red) and 45*S* rDNA (green) exhibiting one multivalent and one bivalent highlighted by the ellipse with white dashes that can be scored. (D) Four bivalents from A, with diagrammatic representations of homologous chromosomes in different shades of blue physically linked by chiasmata, shown by white crosses. (E) Histogram of the frequency of meiotic stability in individual plants from all populations. (F) Comparison of chiasmata position and frequency of *A*. *lyrata* bivalents from diploid (red), stable tetraploid (green) and, unstable tetraploid (blue). (G) Representative image of HEI10 foci at late pachytene in a stable nucleus, immunolocalised with SMC3 and counterstained with DAPI. (H) Representative image of HEI10 foci at late pachytene in an unstable nucleus, immunolocalised with SMC3 and counterstained with DAPI. (I) HEI10 counts from stable and unstable pachytene nuclei. Bars for A-C, G and H = 10μm.

A thorough examination of meiotic stability was performed on 52 plants obtained from individual maternal seed lines sampled from tetraploid populations covering a range of known genomic backgrounds and demographic histories from the Wachau region and east Austrian Forealps. Relative genomic contributions (proportions of admixture) from *A*. *lyrata* and *A*. *arenosa* from sampled populations had previously been determined [25]. Sampled populations were: LIC, MAU, MOD, PIL, SCB, KAG, ROK (*A*. *lyrata* and *A*. *lyrata*-like hybrids that contain >50% genomic contribution from *A*. *lyrata*) and, TBG, SEN and WEK (*A*. *arenosa*/*A*. *lyrata* introgressants that contain >50% genomic contribution from *A*. *arenosa*) (Fig 1). Meiotic stability was assessed in individual plants by performing cytological analyses on metaphase I (MI) chromosome spreads of pollen mother cells (PMCs). A rod bivalent forms when only one chiasma (the cytological manifestation of a CO) connects a homologous chromosome pair (Fig 2A and 2D1–2D3). A ring bivalent forms when chiasmata occur in both chromosome arms of homologous pairs (Fig 2A and 2D4). Quadrivalents are structures formed of four chromosomes, usually two pairs of homologous chromosomes and, multivalents form between multiple chromosomes either connected by chiasmata or interlocks (Fig 2C). As a control, chiasma frequency and distribution were scored at MI in diploid *A*. *lyrata* PMCs (Fig 2A). For the tetraploids, MI nuclei were scored as stable when 16 individual bivalents could be observed aligned on the MI plate and unstable if quadrivalents or multivalents were observed (Fig 2B and 2C and S1 Fig). For each maternal line we scored blind the percentage of stable versus unstable nuclei per plant, revealing a range from 20–100% (Fig 2E). Furthermore, we scored chiasmata as distal, interstitial or proximal to the centromere based on chromosome bivalent shapes in sampled populations (8 x diploid plants, 5 x predominantly stable LIC plants and, 2 x KAG and 2 x MAU predominantly unstable plants) (Fig 2D1–2D4). In unstable nuclei, only a proportion of individual bivalents could be scored per nucleus (ranging from 1–11 per cell), that were not associated with other chromosomes (Fig 2C, dashed ellipse). A FISH analysis utilizing the 5*S* and 45*S* rDNA probes revealed that all chromosomes that could be scored in the samples of unstable nuclei were observed either as individual bivalents or associated with multivalents. From the total 16 pairs of chromosomes per nucleus, seven did not hybridize with the 5*S* and 45*S* rDNA probes, five with the 5*S* only, two with 45*S* only, and two with both 5*S* and 45*S*. However, the FISH analysis revealed a chromosome bias for those that could be scored in the unstable nuclei with an underrepresentation of chromosomes containing the 45*S* rDNA nucleolar organizing regions (NOR) and an overrepresentation of chromosomes without the 45*S* rDNA (Table 1). A Chi-squared test revealed that these values were significantly different (χ_[3]_^2^ = 9.54, *P* < 0.05), indicating that chromosomes with the 45*S* rDNA NOR were more likely to form multivalents (Fig 2C).

**Table 1:** Frequencies of expected and observed bivalents that were scored for chiasmata in unstable nuclei labelled with ribosomal DNA FISH probes.

	Bivalents with no rDNA FISH probes	Bivalents with 5*S* probe only	Bivalents with 45*S* probe only	Bivalents with 5*S* and 45*S* probe
**Expected**	34	24	9.5	9.5
**Observed**	46	21	6	4

All bivalents from stable cells were grouped into a set of 16, however only a proportion of bivalents from unstable cells could be scored. Therefore, these were randomly grouped into a set of 16 pseudo-nuclei with the caveat that these were less likely to form multivalents. Overall, significantly more chiasmata were observed in diploid *A*. *lyrata* MI bivalents than those from tetraploid stable or unstable nuclei (1.52±0.3, n = 312; versus 1.12±0.2, n = 960 and 1.26±0.3, n = 590, respectively, Mann Whitney Test, P < 0.001). The frequency of distal chiasmata was not significantly different between bivalents in diploid and stable tetraploid nuclei but was reduced in bivalents from unstable tetraploid nuclei (Fig 2F). Bivalents from stable tetraploid nuclei had significantly fewer interstitial and proximal chiasmata compared to bivalents from diploids and those from unstable nuclei, whereas interstitial and proximal chiasmata were not significantly different between bivalents from diploids and unstable tetraploid nuclei (Fig 2F).

A HEI10 immunocytological analysis was performed at late pachytene to confirm whether there was a difference in CO frequency between stable and unstable nuclei (Fig 2G–2I). MAU8.11 (phenotyped at MI with 98% stability) and SEN2.2 (phenotyped at MI with 21% stability) were selected as extreme examples of meiotic stability (S1 Table). During pachytene HEI10 marks class I CO sites that ensure each chromosome pair receives at least one ‘obligate’ CO/chiasma, and accounts for ~85% of all COs [30]. In our analysis HEI10 also marked heterochromatic DNA, which was not scored as designated class I CO sites. In MAU8.11 nuclei, an average of 20.4 HEI10 foci per pachytene (n = 30) were scored and SEN2.2 nuclei, an average of 22.5 HEI10 (n = 30) were scored, revealing that the predominantly unstable nuclei had significantly greater numbers of HEI10 foci (Wilcoxon rank sum test, P<0.05) (Fig 2I). Stable nuclei contained an average of 1.28 HEI10 foci/bivalent, whereas the predominantly unstable nuclei contained an average of 1.4 HEI10 foci/bivalent.

### Association of haplotypes with meiotic stability in *A*. *lyrata*/*A*. *arenosa* tetraploids

**Fig 3:**
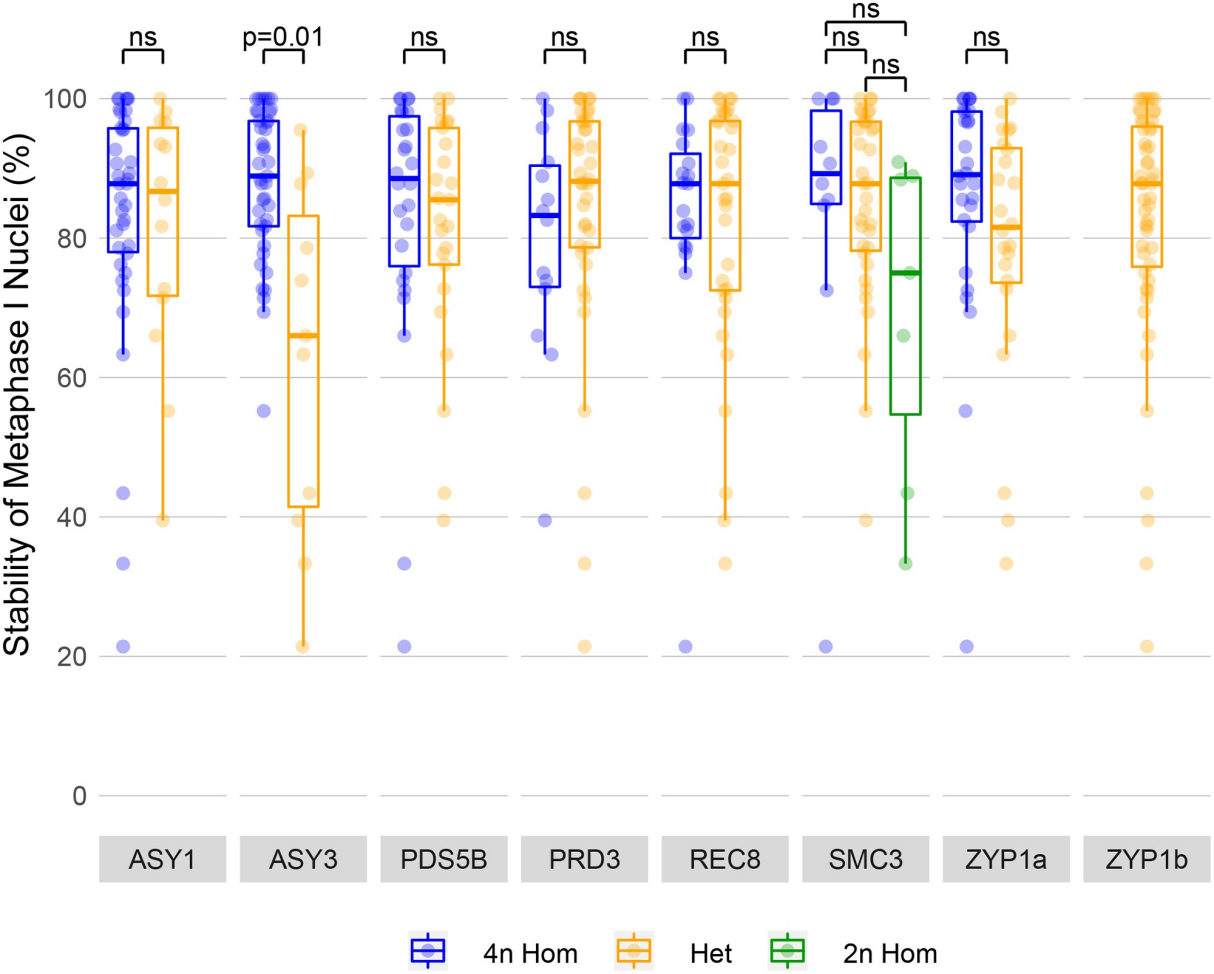
Association analysis of meiotic chromosome stability at metaphase I with meiosis gene haplotypes. Individual plants are collapsed into groups containing either homozygous 4n derived (blue), 2n/4n heterozygous (orange) or 2n homozygous ancestral (green) haplotypes from all populations tested. Each data point represents the male meiotic stability of an individual plant for each specific haplotype.

The 52 tetraploid plants phenotyped for male meiotic stability were then genotyped for the proportion of each meiosis gene haplotype by high-throughput sequencing. Accurate genotyping required obtaining precise population reference sequences from published genomic data from these populations [25]. Degenerate primers were used to amplify full length gene amplicons of the eight meiosis genes from the 52 tetraploid plants (including exons and introns) for construction of Nextera LITE libraries. Libraries were barcoded per plant and sequenced by MiSeq, generating an average sequence depth across all loci of >2000x, from which we determined the proportion of each haplotype per plant by SNP frequency. Because the coding regions of all eight meiosis genes from representative populations were cloned and Sanger sequenced (described below in ‘Adaptive polymorphisms in meiosis genes’), it was possible to resolve individual haplotypes. As these plants were drawn from a diversity of wild populations it was not surprising that an average of 7.25 alleles were identified for each of the eight meiosis genes. Given the limited sample size of 52 individuals it was not possible to statistically interrogate associations between all individual haplotypes for the eight genes. Consequently, similar haplotypes were collapsed together and classified into two groups: haplotypes with derived tetraploid alleles, and those with ancestral diploid alleles, as determined through phylogenetic analysis (S2–S5 Figs). Derived tetraploid haplotypes were those possessing conserved polymorphisms relative to the diploid reference sequences. For example, with *Asynapsis3 ASY3*, phylogenetic analysis identified three derived alleles (*TD1*, *TD2* and *TD3*) that were very highly related and clearly different from the diploid alleles (S2 and S4 Figs). For each gene (except *ZYP1b*, which was heterozygous in all populations tested) a large proportion of the individuals carried four derived tetraploid haplotypes, whilst the others carried a mixture of derived tetraploid and diploid haplotypes. We therefore tested whether the presence of diploid haplotypes influenced meiotic stability. To do this, we classified the allele state at each of the eight meiosis genes in each individual tetraploid as either homozygous (i.e. exclusively either derived tetraploid haplotypes, or alternatively ancestral diploid haplotypes) or heterozygous (individuals harbouring both ancestral and derived alleles together at a given locus), and tested for any associations between these genotypes and meiotic stability by cytological analysis (Bonferroni corrected pairwise Mann-Whitney-Wilcoxon; S1 Table). This revealed that only the meiotic chromosome axis gene *ASY3* had a significant effect on meiotic stability (Fig 3). Plants that were heterozygous for the *ASY3* ancestral diploid haplotype and the derived tetraploid haplotype had significantly more unstable male metaphase I nuclei than those in plants homozygous for the derived *ASY3* haplotype (4n Hom $\tilde{x}=88.9$, IQR = 15.1, n = 41, $\tilde{x}=66$, IQR = 41.7, n = 11, p = 0.008). There was a large range of meiotic stability within the *ASY3* heterozygotes, so smaller effects from the other seven meiosis genes cannot be excluded. For example, there was a trend for the ancestral diploid *SMC3* allele to associate with lower meiotic stability than the tetraploid homozygotes or diploid/tetraploid allele heterozygotes, although sampling sizes were not great enough to statistically confirm this trend. In addition, MiSeq sequencing revealed that the diploid *ASY3* allele in the PIL population had an amino acid substitution near the N-terminus that may have had an effect on meiotic stability.

### Adaptive polymorphisms in meiosis genes

**Fig 4:**
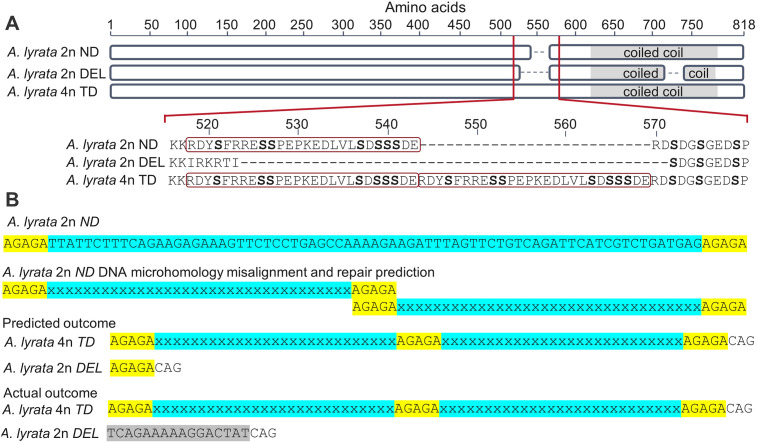
Structural variants of the three major *A*. *lyrata ASY3* alleles. (A) The serine-rich region (red box) with serines highlighted in bold in the non-duplicated (ND) ancestral diploid allele, absence in the deletion (DEL) diploid allele and tandemly duplicated (TD) in the derived tetraploid allele possessing two serine-rich regions (red boxes) as well as putative SUMO sites at K517, K531 and K556. (B) Major structural variation at the *ASY3* locus suggesting that misalignment by DNA microhomology (highlighted yellow) and recombinational repair between the ancestral *ND* alleles may have led to the formation of the *TD* allele (blue = region of duplication). The outcome does not quite match the prediction as there are bases of unknown origin in the *DEL* allele (shaded in grey), but formation of these alleles may be independent events. The x’s highlighted in blue represent bases between the AGAGA sites as shown for *A*. *lyrata* 2n *ND*.

Previous studies have detected polymorphic amino acids in meiosis genes between diploids and tetraploids in *A*. *arenosa* and *A*. *lyrata* by aligning short read sequences to the *A*. *lyrata* reference, but could not infer contiguous autotetraploid alleles [8, 25, 31]. To overcome this and resolve individual haplotypes, we amplified, cloned and Sanger sequenced the coding regions of the eight meiosis genes from diploid *A*. *arenosa* SNO and *A*. *lyrata* PER populations, tetraploid *A*. *arenosa* SEN, TBG and WEK populations, as well as *A*. *lyrata* KAG and MAU populations. This approach provided high-resolution sequence polymorphism data for a total of 320 cDNA transcripts and by incorporating MiSeq data from all populations we were able to resolve 58 alleles with an average of seven alleles for each of the eight meiosis genes, thus allowing us to detect both structural variation including indels and divergent SNP variation (S6–S13 Figs, S2–S4 Tables, Dryad Digital Depository (https://doi.org/10.5061/dryad.x69p8czfb)). The eight encoded proteins associated with the synaptonemal complex are conserved at the secondary and tertiary amino acid level, rather than at the primary sequence [32]. Phosphorylation of synaptonemal complex proteins is known to be important for their activity [33][34], so software was used to determine if the identified polymorphisms may affect amino acids with important phosphorylation sites. Overall, 45% of polymorphisms led to either gains or losses of putative serine/threonine phosphorylation sites and 55% were non-phosphorylation sites. The ASY3 derived adaptive allele and ZYP1b exhibited the greatest quantity of residue changes compared to the diploids that were conserved in tetraploid populations (45 and 44, respectively) (S14 Fig), whilst SMC3 had none (although there were population specific SMC3 SNPs). Of the 45 polymorphic residues between the ancestral *A*. *lyrata* diploid ASY3 allele and the derived tetraploid ASY3 allele, 27 were due to a tandem duplication (TD allele) in a serine-rich region of the protein. The three ASY3 alleles (TD1, TD2 and TD3) all share this same tandem duplication, although population specific polymorphisms also occur outside this region (S6 Fig). The serine-rich region is upstream of the coiled-coil domain, possessing putative ATM/CKII phosphorylation motifs and a predicted SUMO site (K556, GPS-SUMO), and the TD allele contains 14 serines in this region, compared to 7 in the ancestral *A*. *lyrata* non-duplicated allele (ND) (Fig 4A). As the *ASY3 TD* allele had highest sequence similarity to the diploid *A*. *lyrata ASY3 ND* allele, to investigate the provenance of the adaptive tandem duplication, we exhaustively screened the local diploid *A*. *lyrata* population (PER) geographically adjacent to the *A*. *lyrata* autotetraploid LIC and MOD populations (Fig 1). 128 plants from the diploid *A*. *lyrata* PER population were screened, but the *ASY3 TD* allele was not identified, indicating the current absence (or vanishingly low frequency) of the *TD* allele, although it did reveal the presence of a deletion (*DEL*) allele at 7% frequency, where the entire serine-rich region is absent. We therefore cloned and sequenced genomic DNA from *ASY3 ND*, *TD* and *DEL* and aligned them. This revealed a 78 bp region of exon 2 is duplicated in-frame in the *ASY3 TD* allele that is missing in the *ASY3 DEL* allele between two AGAGA sites that possess DNA microhomology. We speculate that this DNA microhomology may have been instrumental in the formation of the *ASY3 TD* allele by homologous DNA repair through a replication error or during meiotic recombination (Fig 4B).

### Meiosis gene flow between A. lyrata/A. arenosa tetraploid populations

**Fig 5:**
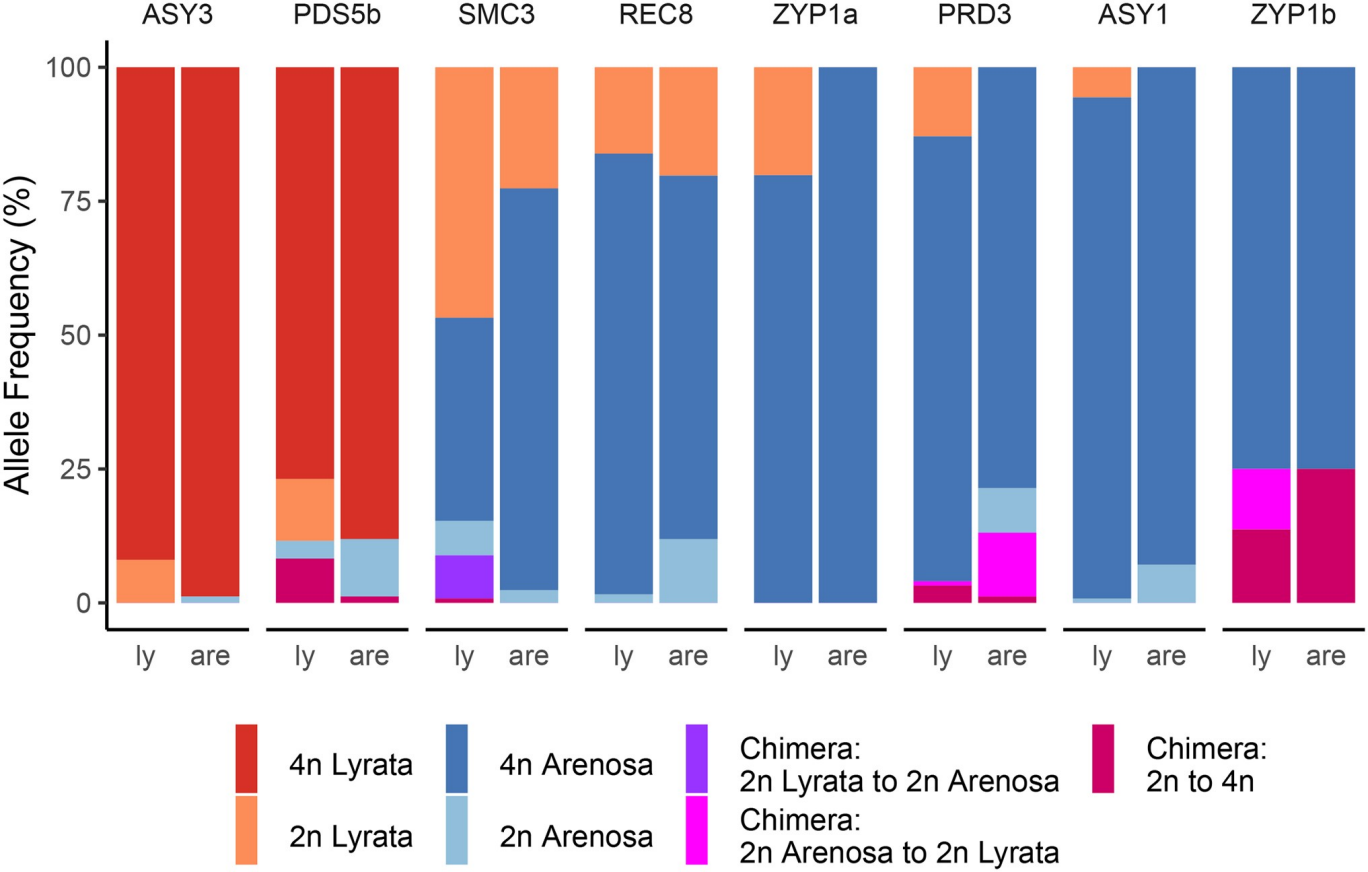
Contrasting origins and polymorphisms of adaptive meiosis gene haplotypes in tetraploid introgressants of *A*. *lyrata*/*A*. *arenosa*. Haplotype frequencies of adaptive meiosis alleles from *A*. *lyrata* (*Ly*) and *A*. *arenosa* (*Ar*) populations.

We categorized the admixture proportions of the tetraploid populations into predominantly *A*. *lyrata* (LIC, MOD, SCB, KAG, PIL and ROK) or predominantly *A*. *arenosa* (WEK, SEN and TBG), based on a demographic analysis [25]. The Sanger sequenced coding regions of the eight meiosis genes provided high quality reference data to determine allelic origin and to infer the direction of gene flow. Maximum likelihood and Bayesian phylogenetic analyses showed that each of these gene sequences clustered cleanly according to ploidy (S2–S5 Figs). The ancestral diploid sequences were further divided into *A*. *lyrata* or *A*. *arenosa*. Tetraploid sequences clustered into separate alleles irrespective of species, although population differences persisted (S2–S5 Figs). The *ASY3 TD* allele that associates with meiotic stability in tetraploids clusters with the *A*. *lyrata* diploid sequence (S2 and S4 Figs), as does *PDS5b* (S2 and S4 Figs). Conversely, derived tetraploid alleles for *ASY1*, *PRD3*, *REC8*, *SMC3*, *ZYP1a* and *ZYP1b* had highest homology with diploid *A*. *arenosa* (S2 and S4 Figs). The *A*. *lyrata ASY3 TD* allele is present at 99% frequency in tetraploid *A*. *arenosa* populations tested and 95% in tetraploid *A*. *lyrata* populations (Fig 5). The analysis revealed a small number of ancestral diploid *A*. *arenosa* and *A*. *lyrata* alleles in these populations. In contrast, the *ASY1* allele derived from diploid *A*. *arenosa* had a frequency of 94% in *A*. *arenosa* tetraploid and 93% in *A*. *lyrata* tetraploid, indicating bidirectional gene flow (Fig 5). More surprisingly, bidirectional gene flow between the ancestral *SMC3* and *REC8* diploid alleles was observed in the tetraploids (Fig 5).

### Widespread chimerism in meiosis gene alleles

Our analysis of Sanger and MiSeq data identified a novel chimeric allele of *ZYP1b* in all tetraploid populations; chimeric *PRD3* in SCB, SEN and WEK; and chimeric *PDS5b* in ROK. While rare chimeras could potentially result from PCR artefacts [35], these alleles have the same breakpoints in multiple individuals which suggests alterations in the genomic DNA through homologous DNA repair *in planta* [36]. At the 3’ end of all *ZYP1b* tetraploid alleles we detect evidence for a 1474 bp gene conversion (GC) to *ZYP1a* (S7 Fig). There is also evidence of GC in *PRD3* between *A*. *arenosa* and *A*. *lyrata* ancestral diploid alleles (S5 Fig). In 11 plants from *A*. *arenosa* and *A*. *lyrata* populations, the first 740 bp of the diploid *PRD3* allele is more similar to the diploid *A*. *arenosa* than to *A*. *lyrata* (7 vs 21 SNPs), while the remaining 625 bp of coding sequence has a higher similarity to diploid *A*. *lyrata* than to *A*. *arenosa* (5 vs 24 SNPs). In *PDS5b*, at the 5’ end of tetraploid alleles from five *A*. *arenosa* plants we observe evidence for a GC of the first two exons to the diploid allele, providing evidence of GC (or CO) between ploidy levels (S5C Fig). In addition, analysis of genome resequencing data [25] revealed a 3’ GC from a diploid *ASY1 A*. *lyrata* allele to a tetraploid *A*. *arenosa* allele in the KAG population (S5D Fig). The apparent widespread presence of gene conversion products in these loci suggests a mechanism by which genes may be modified in a narrow genetic window to confer adaptative advantages.

## Discussion

Here we aimed to determine the impact of strongly selected meiosis gene alleles that underwent recent selective sweeps on the rapid evolution of autotetraploid meiotic stability in *A*. *lyrata/A*. *arenosa hybrids* and introgressants and to trace their evolutionary origin. By associating genotypic and cytological phenotypic data our analysis suggests that *ASY3* is the major locus currently stabilising autotetraploid male meiosis in these populations. We identified structural variation of meiosis alleles including a novel derived *ASY3* allele with a tandem duplication (*TD*) in a serine-rich region of the protein that is associated with stable meiotic chromosome phenotypes in the tetraploids, as well as novel *ASY1*, *PDS5b*, *PRD3* and *ZYP1b* chimeric alleles originating between diploid and tetraploid *A*. *arenosa* and *A*. *lyrata*.

A cytological metaphase I (MI) analysis revealed that chiasmata in the autotetraploids were significantly reduced in both stable and unstable nuclei compared to diploid *A*. *lyrata*. Moreover, chiasma frequencies in meiotically stable nuclei were significantly reduced in regions proximal and interstitial to the centromere. A shift in chiasma distribution may reflect a fundamental mechanism for meiotic adaptation to autopolyploidy [37]. The unstable nuclei occur due to unregulated meiotic recombination between multiple chromosomes, either homologous or non-homologous. All chromosomes that could be distinguished were present in multivalents, although there was a bias for the 45*S* rDNA containing chromosomes to occur more frequently, which may be due to the NORs clustering during prophase I, thus bringing non-homologous chromosomes into close proximity. A reduction in numbers of chiasmata in stable nuclei was supported by an immunocytological approach counting HEI10 foci that mark designated class I COs. HEI10 foci numbers were significantly lower in pachytene nuclei from a stable plant (20.4 HEI10 foci per cell) compared to nuclei from the most unstable plant (22.5 HEI10 foci per cell)(P<0.01). This contrasts to autotetraploid *A*. *arenosa* where HEI10 counts in plants homozygous for *ASY1* or *ASY3* diploid ancestral alleles were not significantly different to plants homozygous for the derived alleles [29]. However, our analysis utilised genotypes with extreme meiotic stabilities. In [29], MI meiotic instability was less extreme, possibly reflecting more homogeneity of derived allelic combinations of *PDS5b*, *PRD3*, *REC8*, *SMC3*, *ZYP1* and *ZYP1b* in plants tested.

Our genotype-phenotype association study revealed that among these eight meiosis genes, the allele state of the structurally variable meiotic chromosome axis protein ASY3 was the major factor associating with stable nuclei. Our data also indicated that *REC8* and *SMC3* may have had additive effects, but sample size was not great enough for statistical significance. We hypothesise that the ancestral diploid *ASY3 ND* allele promotes high levels of proximal and interstitial chiasmata, but in a tetraploid background it acts dominantly over the evolved *ASY3 TD* allele, promoting interstitial and proximal chiasmata as well as complex chromosome structures including multivalents. Such multivalents have previously been observed in diploid *A*. *thaliana ZYP1*^*RNAi*^ lines where the authors postulated that chiasmata may have formed between extensive duplications with high sequence similarity on non-homologous chromosomes [23]. The tandemly duplicated serine-rich region in the ASY3 TD allele may function in a manner similar to the budding yeast N-terminus MSH4 degron that destabilizes the protein until it is phosphorylated [38]. Further studies are required to determine if the serine-rich region destabilizes the ASY3 protein thus creating a hypomorphic variant or whether these sites are phosphorylated. The analysed brassica SC phosphoproteome [34] did not recover peptides for ASY3 in the serine-rich region, although similar (serine-aspartic acid) residues recovered from ASY1 were phosphorylated. However, in a recent analysis of the *A*. *thaliana* proteome, Serine 526 of the ASY3 serine-rich region was phosphorylated [39]. The ASY3 TD allele also contains 19 derived residues outside the serine-rich region, of which 10 are predicted serine/threonine phosphorylation site gains or losses, that cannot be ruled out as functionally important along with unknown phosphorylation-related *trans* effects. *Cis*-regulatory variation outside the coding region in ASY3 could also contribute to effects on meiotic stability. Chiasmata are distalized in *A*. *thaliana* chromosome axis mutants *asy1*, *asy3* and *asy4*, presumably due to telomere proximity enabling sufficient inter-homolog pairing, whereas a complete meiotic axis is required to promote high levels of recombination between spatially separated regions in nuclei along the chromosome arms [19, 20, 40]. We hypothesise that the ASY3 TD protein may be hypomorphic and act similarly to *asy1*, *asy3* and *asy4* mutants in distalizing chiasmata [19, 20, 37]. However, when heterozygous with the dominant ancestral ASY3 ND allele, rates of inter-homolog and non-homologous recombination increase. Axis components that favour interstitial and proximal recombination in diploids may promote associations with non-homologous chromosomes in the tetraploids, especially in regions with high sequence homology. However, for stable bivalents once a distal CO site is designated, CO interference may prevent further COs forming.

Phylogenetic analysis revealed that the *ASY3 TD* allele most likely originated from diploid *A*. *lyrata*, consistent with [25]. A screen of the diploid *A*. *lyrata* population geographically adjacent to the tetraploids for the *ASY3 TD* did not detect the allele, but instead identified one with the same region deleted (*DEL*). The *ASY3 DEL* allele coding region is in-frame and contains a second deletion in the coiled-coil. It may be a coincidence that the same region is lost and gained in the *ASY3* alleles, or that this sequence is more susceptible to DNA replicative errors. The region of interest is flanked by DNA microhomology (AGAGA) that is positioned at the putative exchange points and may have led to DNA polymerase slippage or aberrant replication fork repair. It is unlikely to have occurred during meiotic recombination due to the low level of sequence homology. However, we observe numerous examples of apparent gene conversion that appear to have arisen during meiotic recombination in *ASY1*, *PRD3*, *PDS5b* and *ZYP1a/b* between both species and ploidies. As chimeric alleles are present at high frequencies and have single origins, we hypothesise that these polymorphisms are under positive selection. We were unable to test the effect of *ZYP1b* on meiotic stability as there were no homozygotes in our sampled populations. This is surprising and may reflect that another *ZYP1b* locus may have arisen when the apparent gene conversion occurred. When we cross *ASY3 ND*/*DEL* diploid *A*. *lyrata* plants, in the progeny we only recover the same genotype as the parents, suggesting that another locus may have arisen that has not been mapped. The bidirectional gene flow between *A*. *arenosa* and *A*. *lyrata* tetraploids has enlarged the gene pool for beneficial alleles to be borrowed and selected upon and the novel gene converted chimeric alleles may precisely coalesce advantageous sequences from differing origins for adaptation, although this would require further testing with a larger sample size.

Evidence suggests that the origin of the adaptive *ASY3 TD* allele in the tetraploid populations is relatively recent but has spread extensively and introgressed within tight boundaries in the *A*. *lyrata*/*A*. *arenosa* hybrid genomes tested [25]. Our current analysis provides haplotype-level sequence data that supports this hypothesis. The orthologous diploid *ASY3* alleles from *A*. *arenosa* and *A*. *lyrata* are highly divergent, and yet the tetraploid *ASY3 TD* allele is very similar among tetraploid populations. We speculate that preceding the origin of the *ASY3 TD* allele, gene flow of adaptive alleles from *A*. *arenosa* (*ASY1*, *PRD3*, *REC8*, *SMC3*, *ZYP1a* and *ZYP1b*) may have been necessary to establish meiotic stability in the nascent *A*. *lyrata* tetraploids, but has since been relaxed due to the presence of the predominant *ASY3 TD* allele.

The *A*. *lyrata* autotetraploid populations contain plants with variable levels of meiotic stability as well as relatively high frequencies of diploid-like alleles. The diploid alleles may persist due to: a) continuous gene flow via unreduced gametes [41]; b) being beneficial in certain environmental conditions e.g. high altitudes [42]; c) being advantageous during female meiosis (COs are reduced in *A*. *thaliana* female compared to male) [43]; d) inability to purge genetic load in autotetraploids [44] and; e) a limited effect on overall male pollen fecundity, due to an excess of grains transmitted, despite variable quality.

Taken together, our data indicate multiple mechanisms for rapid meiotic evolution in autotetraploid *A*. *lyrata*. They reveal an association between MI stability and a duplication in the serine-rich region in the ASY3 TD allele. Furthermore, tetraploid *A*. *lyrata* has introgressed *ASY1*, *PRD3*, *REC8*, *SMC3*, *ZYP1a* and *ZYP1b* alleles from *A*. *arenosa* by gene flow. Finally, novel chimeric genes of *ASY1*, *PDS5b*, *PRD3*, *ZYP1a* and *ZYP1b* have arisen evidently through gene conversion, suggesting highly dynamic mechanisms to generate variation that may be selected upon during evolution to ensure meiotic success in these populations.

## Material and methods

### Cloning and sequencing of meiosis gene transcripts

Plants were grown from seed to obtain fresh flower buds from diploid *A*. *arenosa* SNO and *A*. *lyrata* PER populations, and tetraploid *A*. *arenosa*, SEN, TBG and WEK and *A*. *lyrata* KAG and MAU populations [25]. These buds were collected, flash frozen in liquid nitrogen and stored at -80C until RNA extraction. Total RNA was extracted using a Bioline ISOLATE II RNA Plant Kit (Bioline Ltd, London, UK), following manufacturer’s instructions, eluting into a final volume of 60 μl nuclease free water. Concentration and purity were determined using a NanoDrop spectrophotometer (LabTech International, Lewes, UK) and one microgram of total RNA was electrophoresed on a non-denaturing 1% (w/v) agarose gel to check for degradation. First strand cDNA was reverse transcribed from 0.5 μg of total RNA using a Quantitect Reverse Transcription Kit (Qiagen, Hilden, Germany) that incorporates genomic DNA removal prior to reverse transcription. The coding regions of 8 meiosis genes (*ASY1*, *ASY3*, *PRD3*, *PDS5b*, *REC8*, *SMC3*, *ZYP1a* and *ZYP1b*) were amplified by PCR using 0.2 μM primers (S5 Table) designed using HiSeq data [45] and Platinum *Taq* DNA Polymerase High Fidelity (ThermoFisher Scientific, MA, USA). PCR conditions were as follows: 94 °C for 2 min, followed by 35 cycles of 94 °C for 15 s, 60–65 °C for 30 s and 68 °C for 2–5 min (see S5 Table), with a final extension of 68 °C for 5–10 min. PCR products were electrophoresed on a 2% (w/v) agarose gel, and single bands of the expected size were excised and purified with a Monarch DNA Gel Extraction kit (New England Biolabs, MA, USA). Purified PCR products were cloned into pCR-XL-TOPO vector using a TOPO XL PCR Cloning Kit (ThermoFisher Scientific) following the manufacturer’s instructions. For each gene a total of 8 clones from each plant were isolated from overnight LB cultures using an ISOLATE II Plasmid Mini Kit (Bioline) prior to sequencing with universal M13F and M13R primers by GATC Biotech (Konstanz, Germany). Nucleotide sequences of the cDNAs were processed in Geneious 11.1.2 (https://www.geneious.com) to remove vector and low-quality sequence before using BLASTN to search the June 2010 (v.1.0/INSDC) assembly of the North American *A*. *lyrata* reference genome [46] and NCBI nonredundant (nr) database for confirmation that the obtained cDNAs were the expected gene transcripts. Primer walking was then used to sequence the entire length of the transcript. For each meiosis gene, cDNAs from each population were aligned with the respective Ensembl gene predictions from the *A*. *lyrata* reference genome (S6 Table), and to act as outgroups, *A*. *thaliana* transcripts obtained from The Arabidopsis Information Resource (TAIR) using the MUSCLE 3.8.425 plugin in Geneious 11.1.2 with default settings [47]. Phylogenetic trees were constructed using a maximum likelihood (ML) method with PhyML v3.3 [48] and bootstrap testing (1000 replicates). The ML phylogenetic trees were confirmed by a Bayesian approach with MrBayes v3.2.6 [49] with 1.1 x 10^6^ generations of Markov Chain Monte Carlo searches containing 4 heated chains, a heated chain temperature of 0.2 and a burn-in of 100,000 generations. MrBayes trees were subsampled every 200 generations and the confidence values presented as posterior probabilities. The best nucleotide substitution method for both ML and MrBayes was determined with the Find Best-Fit Substitution Model in Mega v10.0 [50].

### Meiotic haplotype genotyping

Genomic DNA was extracted from leaf material of each of the 52 plants in the study using a DNeasy Plant Mini Kit (Qiagen) and eluting into 100 μl nuclease free water. Full length coding regions of each of the 8 meiosis genes (including introns) were amplified from this genomic DNA by PCR using Platinum SuperFi Green PCR Master Mix (ThermoFisher Scientific) and 0.5 μM primers (S5 Table) designed against the Sanger sequenced cDNA of the 8 meiosis genes (see above). PCR conditions were as follows: 98 °C for 30 s, followed by 35 cycles of 98 °C for 30 s, 60–63 °C for 10 s and 72 °C for 2.5–10 min (see S5 Table), with a final extension of 72 °C for 5–10 min. PCR products were electrophoresed on a 1% (w/v) agarose gel, and single bands of the expected size were excised and purified with a Monarch DNA Gel Extraction kit (New England Biolabs). Libraries were constructed using 1ng of input DNA in a Low Input, Transposase Enabled (LITE) pipeline developed at the Earlham Institute (Norwich, UK) and based on the Illumina Nextera kits (Illumina, San Diego, CA, USA) [51]. Each library was constructed using unique 9 bp dual index combinations allowing samples to be multiplexed. Pooled libraries were size selected between 600 and 750 bp on a BluePippin (Sage Science, Beverly, MA, USA) 1.5% Cassette and then sequenced with a 2x250 bp read metric on an Illumina MiSeq sequencer.

MiSeq fastq files were imported into Geneious 11.1.2 (https://www.geneious.com) and R1 and R2 reads paired. Quality trimming of reads was performed with the BBDuk Adaptor/Quality Trimming v.37.64 plugin with default settings (Min quality:20; Min overlap:20; Min length:20). For each of the 52 individuals, trimmed reads were mapped to each of the 8 meiosis genes (S6 Table) from the North American *A*. *lyrata* reference genome [46] using Geneious 11.1.2 Read Mapper (Medium sensitivity; 5 iterations and default settings). SNPs relative to the reference genome genes were then called (Minimum Variant Frequency 0.25; Maximum Variant P-value 6x10^-6^) and used to identify and determine the proportion of 2n and 4n alleles for each gene per plant using a set of allele specific SNPs (as revealed from the Sanger sequencing of meiosis gene transcripts described above). Allele specific indels (eg *ASY3 TD*) were identified by associated SNPs.

### Cloning and sequencing of ASY3 alleles

Genomic DNA was extracted from diploid *A*. *lyrata* PER plants as above and a short section of *ASY3* was PCR amplified using 0.5 μM primers (S5 Table) and MyTaq Red Mix (Bioline). PCR conditions were as follows: 95 °C for 2 min, followed by 35 cycles of 95 °C for 30 s, 69 °C for 30 s and 72 °C for 30 s, with a final extension of 72 °C for 5 mins. PCR products were electrophoresed on a 2% (w/v) agarose gel and the lower band (~125 bp) corresponding to a partial *ASY3 DEL* allele was excised and purified with a Monarch DNA Gel Extraction kit (New England Biolabs). Purified PCR products were cloned into pCR 4-TOPO vector using a TOPO TA Cloning for Sequencing Kit (ThermoFisher Scientific) following manufacturer’s instructions. A total of 12 clones were isolated, purified and Sanger sequenced as above. Nucleotide sequences were processed in Geneious 11.1.2 (https://www.geneious.com) to remove vector and low-quality sequence before aligning with *ASY3 ND*/*TD* transcript alleles as described above. Primers designed against the partial *DEL* sequence were used to obtain the 3’ end of the transcript from 3 μg *ASY3 ND/DEL* heterozygous 2n *A*. *lyrata* (PER) floral bud total RNA using a GeneRacer Kit (ThermoFisher Scientific) following manufacturer’s instructions. Purified PCR products were cloned into pCR4-TOPO vector, sequenced and processed as above.

*ASY3 ND*, *TD* and *DEL* alleles were PCR amplified from genomic DNA extracted from diploid and tetraploid *A*. *lyrata* respectively using 0.2 μM primers designed against *ASY3* cDNA sequences obtained above (S5 Table) and Q5 High-Fidelity DNA Polymerase (New England Biolabs). PCR conditions were as follows: 98 °C for 2 min, followed by 35 cycles of 98 °C for 10 s, 63°C for 30 s and 72 °C for 4 min, with a final extension of 72 °C for 10 min. PCR products were electrophoresed on a 1% (w/v) agarose gel, and single bands of the expected size were excised and purified as above. Purified PCR products were cloned into pDrive (Qiagen) and sequenced by Eurofins Genomics (Ebersberg, Germany).

### Cytology

Chromosome spreads were performed as in [52] [25] on all populations used in this study. The HEI10 immunocytological analysis was performed using the protocol [53] with anti-AtSMC3 rat and anti-AtHEI10 rabbit antibodies described in [54]. Nikon Eclipse Ci and Ni-E microscopes installed with NIS Elements software were used to capture images of chromosomes.

### Protein predictions

Protein post-translational predictions were provided by KinasePhos2.0 [55] and NetPhos3.1 [56] and SUMO sites were predicted by GPS-SUMO [57].

### Statistical analyses and Map drawing

Statistical analysis was performed using the R *Stats* package. Mann-Whitney Wilcoxon tests were performed with function *wilcox*.*test*. Bonferoni adjusted p values were calculated using the function *p*.*adjust*. The map was drawn with ggmap [55] using map tiles by Stamen Design, under CC BY 3.0. Data by OpenStreetMap, under ODbL.

## Supporting information

S1 Fig Examples of DAPI stained male meiotic metaphase I chromosome spreads from tetraploid plants used in this study. Stable (A) and unstable (B). Scale bar = 10μM.
(TIF)

S2 Fig Phylogenetic trees of meiosis genes indicating origin of alleles selected in the autotetraploids constructed using a Bayesian approach with MrBayes v3.2.6. (A) *ASY1*, (B) *ASY3*, (C) *PDS5b*, (D) *PRD3*. Bayesian posterior probabilities are indicated at the internodes of each branch. The dissimilarity scale showing substitutions per nucleotide is located at the bottom of each tree. Diploid and tetraploid alleles are indicated by ‘2’ and ‘4’ respectively (for *ASY3* diploid = ND, tetraploid = TD), while the putative origins are indicated by ‘r’ (*A*. *arenosa*) and ‘y’ (*A*. *lyrata*).
(TIF)

S3 Fig Phylogenetic trees of meiosis genes indicating origin of alleles selected in the autotetraploids constructed using a Bayesian approach with MrBayes v3.2.6. (A) *REC8*, (B) *SMC3*, (C) *ZYP1a/ZYP1b* 5’ end, (D) *ZYP1a/ZYP1b* 3’ end. Bayesian posterior probabilities are indicated at the internodes of each branch. The dissimilarity scale showing substitutions per nucleotide is located at the bottom of each tree. Diploid and tetraploid alleles are indicated by ‘2’ and ‘4’ respectively, while the putative origins are indicated by ‘r’ (*A*. *arenosa*) and ‘y’ (*A*. *lyrata*).
(TIF)

S4 Fig Phylogenetic trees of meiosis genes indicating origin of alleles selected in the autotetraploids constructed using a maximum likelihood approach with PhyML v3.3. (A) *ASY1*, (B) *ASY3*, (C) *PDS5b*, (D) *PRD3*. Maximum likelihood bootstrap values are indicated at the internodes of each branch (1000 replicates). The dissimilarity scale showing substitutions per nucleotide is located at the bottom of each tree. Diploid and tetraploid alleles are indicated by ‘2’ and ‘4’ respectively (for *ASY3* diploid = ND, tetraploid = TD), while the putative origins are indicated by ‘r’ (*A*. *arenosa*) and ‘y’ (*A*. *lyrata*).
(TIF)

S5 Fig Phylogenetic trees of meiosis genes indicating origin of alleles selected in the autotetraploids constructed using a maximum likelihood approach with PhyML v3.3. (A) *REC8*, (B) *SMC3*, (C) *ZYP1a/ZYP1b* 5’ end, (D) *ZYP1a/ZYP1b* 3’ end. Maximum likelihood bootstrap values are indicated at the internodes of each branch (1000 replicates). The dissimilarity scale showing substitutions per nucleotide is located at the bottom of each tree. Diploid and tetraploid alleles are indicated by ‘2’ and ‘4’ respectively, while the putative origins are indicated by ‘r’ (*A*. *arenosa*) and ‘y’ (*A*. *lyrata*).
(TIF)

S6 Fig Nucleotide alignment of *ASY3* in diploid and autotetraploid *A*. *arenosa* and *A*. *lyrata*, in addition to *A*. *thaliana*. The location of diploid and autotetraploid allele specific SNPs are indicated. Coloured bars in each sequence represent base specific SNPs relative to the consensus sequence (Green = A, Blue = C, Black = G, Red = T).
(JPG)

S7 Fig Nucleotide alignments showing examples of putative SC gene conversion-mediated protein polymorphisms. *ZYP1* gene conversion (or CO) between *ZYP1a* (grey) and *ZYP1b* (red) in autotetraploid *ZYP1b* alleles. The location of diploid and autotetraploid allele specific SNPs are indicated. Coloured bars in each sequence represent base specific SNPs relative to the consensus sequence (Green = A, Blue = C, Black = G, Red = T).
(JPG)

S8 Fig Nucleotide alignments showing examples of putative SC gene conversion-mediated protein polymorphisms. Gene conversion between *PRD3* diploid *A*. *lyrata* (green) and *A*. *arenosa* (green) in tetraploid *A*. *arenosa*. The location of diploid and autotetraploid allele specific SNPs are indicated. Coloured bars in each sequence represent base specific SNPs relative to the consensus sequence (Green = A, Blue = C, Black = G, Red = T).
(JPG)

S9 Fig Nucleotide alignments showing examples of putative SC gene conversion-mediated protein polymorphisms. Gene conversion (or CO) between *PDS5b* diploid (yellow) and autotetraploid (green) alleles. The location of diploid and autotetraploid allele specific SNPs are indicated. The location of diploid and autotetraploid allele specific SNPs are indicated. Coloured bars in each sequence represent base specific SNPs relative to the consensus sequence (Green = A, Blue = C, Black = G, Red = T).
(JPG)

S10 Fig Nucleotide alignments showing examples of putative SC gene conversion-mediated protein polymorphisms. Gene conversion (or CO) between *ASY1* diploid *A*. *lyrata* (yellow) and autotetraploid *A*. *arenosa* (green). Coloured bars in each sequence represent base specific SNPs relative to the consensus sequence (Green = A, Blue = C, Black = G, Red = T).
(JPG)

S11 Fig *In silico* translation alignments of meiosis genes. ASY1 (A), ASY3 (B), PDS5b (C), showing conserved amino acid polymorphisms in autotetraploids compared to ancestral diploid alleles. Gains, losses and no change of predicted phosphorylation sites are indicated in blue, yellow and green respectively.
(TIF)

S12 Fig *In silico* translation alignments of meiosis genes. PRD3 (A), REC8 (B), showing conserved amino acid polymorphisms in autotetraploids compared to ancestral diploid alleles. Gains, losses and no change of predicted phosphorylation sites are indicated in blue, yellow and green respectively.
(TIF)

S13 Fig *In silico* translation alignments of meiosis genes. ZYP1a (A) and ZYP1b (B), showing conserved amino acid polymorphisms in autotetraploids compared to ancestral diploid alleles. Gains, losses and no change of predicted phosphorylation sites are indicated in blue, yellow and green respectively.
(TIF)

S14 Fig Summary of conserved amino acid polymorphisms in derived autotetraploid proteins compared to ancestral diploids. The analysis includes gains and losses of predicted serine/threonine phosphorylation sites by KinasePhos2.0 and NetPhos3.1.
(TIF)

S1 Table Genotype and phenotype data. Diploid lyrata alleles = ly; diploid arenosa alleles = ar and ar/ly = diploid arenosa to diploid lyrata putative gene conversions.
(DOCX)

S2 Table Amino acid substitutions conserved in all tetraploids tested relative to diploid *A*. *lyrata* (PER). Putative addition of serine/threonine phosphosites are highlighted in blue and loss of phosphorylation sites highlighted in yellow.
(DOCX)

S3 Table Amino acid substitutions in ASY3 of 2n *A*. *arenosa* (SNO) relative to 2n *A*. *lyrata* (PER). Putative addition of serine/threonine phosphorylation sites are highlighted in blue and loss of phosphorylation sites highlighted in yellow.
(DOCX)

S4 Table Amino acid substitutions conserved in all tetraploids tested relative to diploid *A*. *arenosa* (SNO). Putative addition of serine/threonine phosphorylation sites are highlighted in blue and loss of phosphorylation sites highlighted in yellow.
(DOCX)

S5 Table Primers used for cloning and sequencing. (DOCX)

S6 Table Meiosis genes from *A*. *lyrata* reference genome (DOCX)

S7 Table Data for figures. Raw data used for generating figures.
(XLSX)
