# Supplementary figures and images for: A novel allele of ASY3 is associated with greater meiotic stability in autotetraploid Arabidopsis lyrata
Source: PLoS Genet. 2020 Jul 15;16(7):e1008900. doi: 10.1371/journal.pgen.1008900 (PMC7392332; doi:10.1371/journal.pgen.1008900)

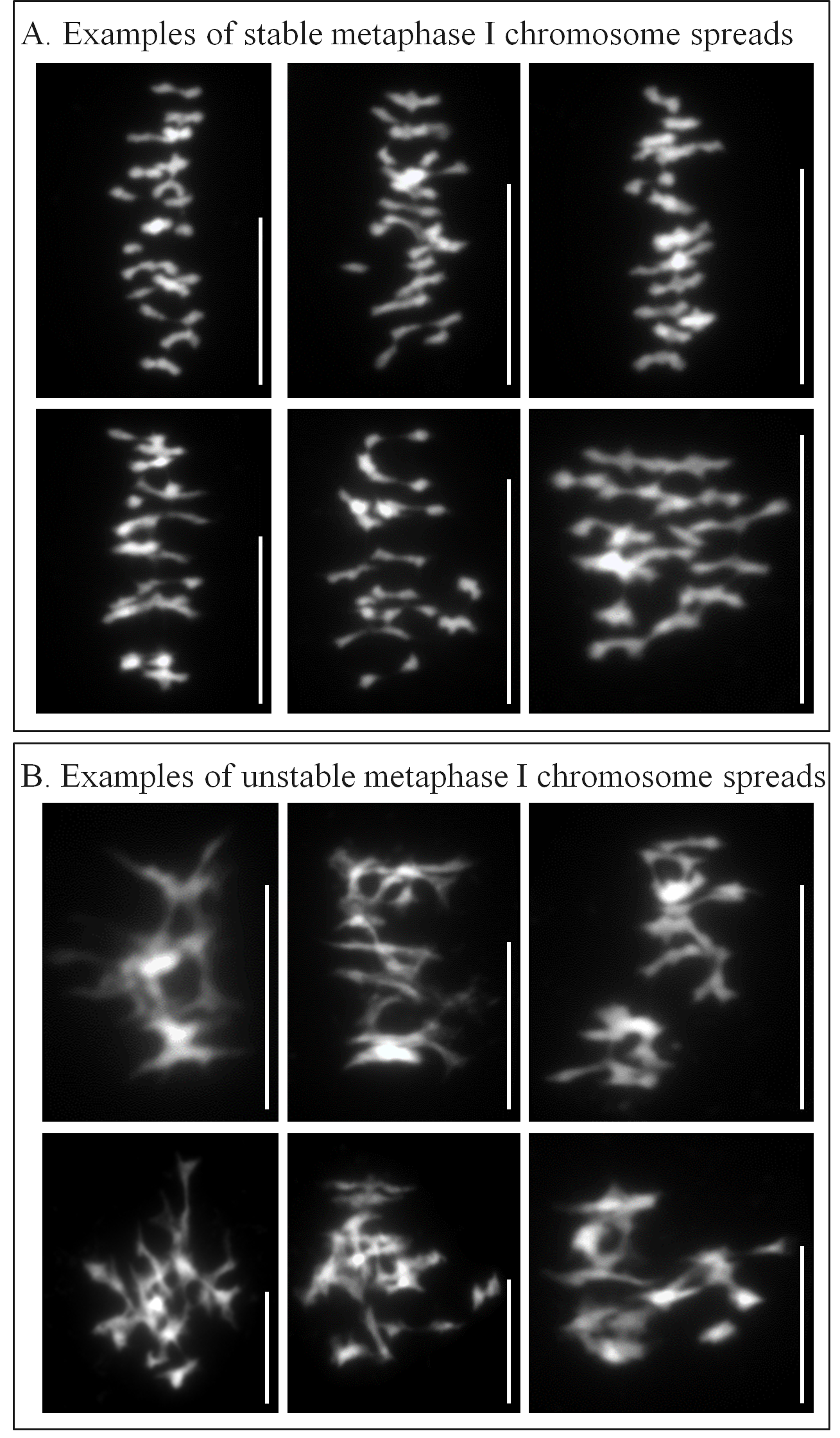

Supplement: S1 Fig — Stable (A) and unstable (B). Scale bar = 10μM. (TIF) [file pgen.1008900.s001.tif]

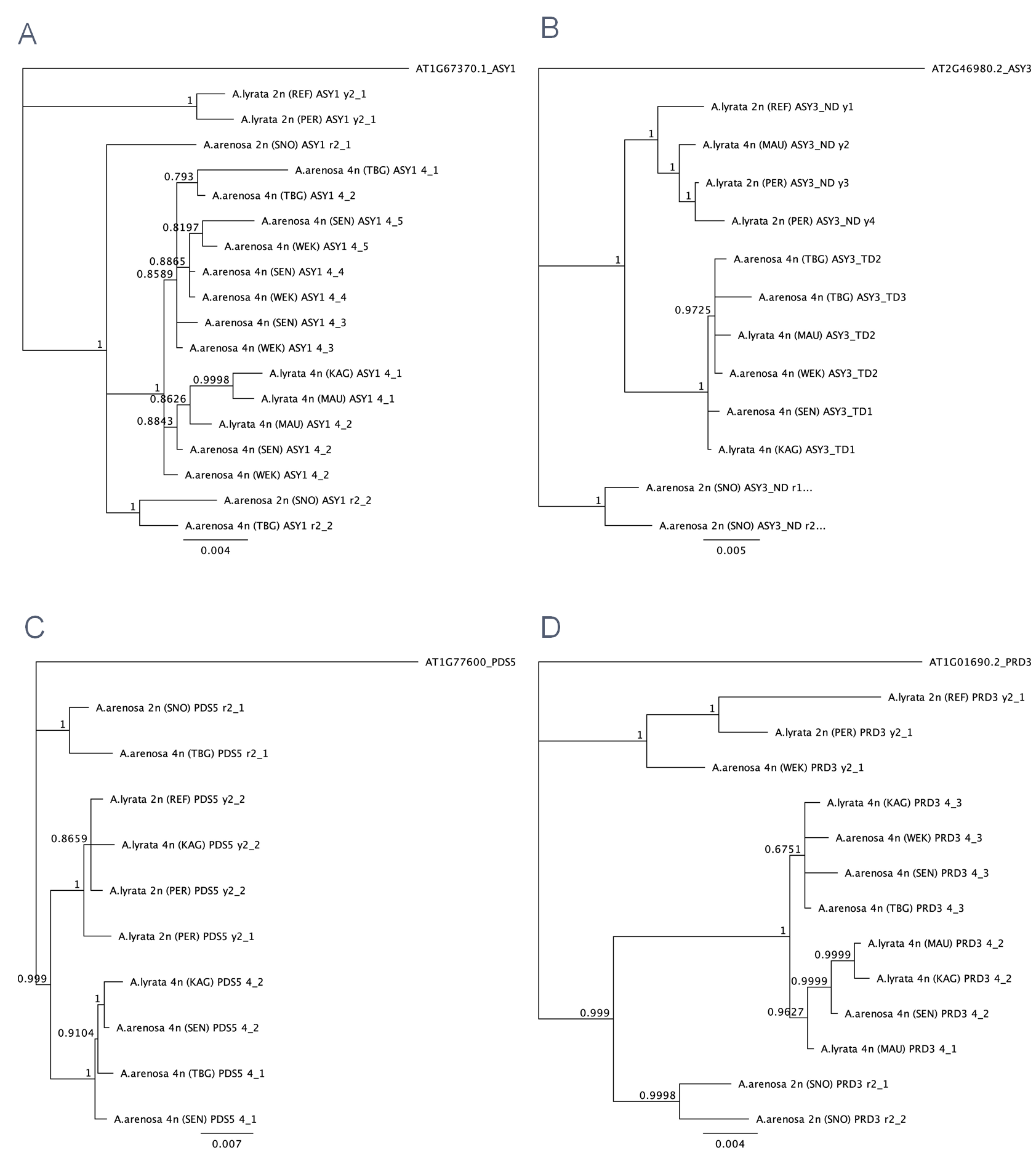

Supplement: S2 Fig — (A) ASY1, (B) ASY3, (C) PDS5b, (D) PRD3. Bayesian posterior probabilities are indicated at the internodes of each branch. The dissimilarity scale showing substitutions per nucleotide is located at the bottom of each tree. Diploid and tetraploid alleles are indicated by ‘2’ and ‘4’ respectively (for ASY3 diploid = ND, tetraploid = TD), while the putative origins are indicated by ‘r’ (A. arenosa) and ‘y’ (A. lyrata). (TIF) [file pgen.1008900.s002.tif]

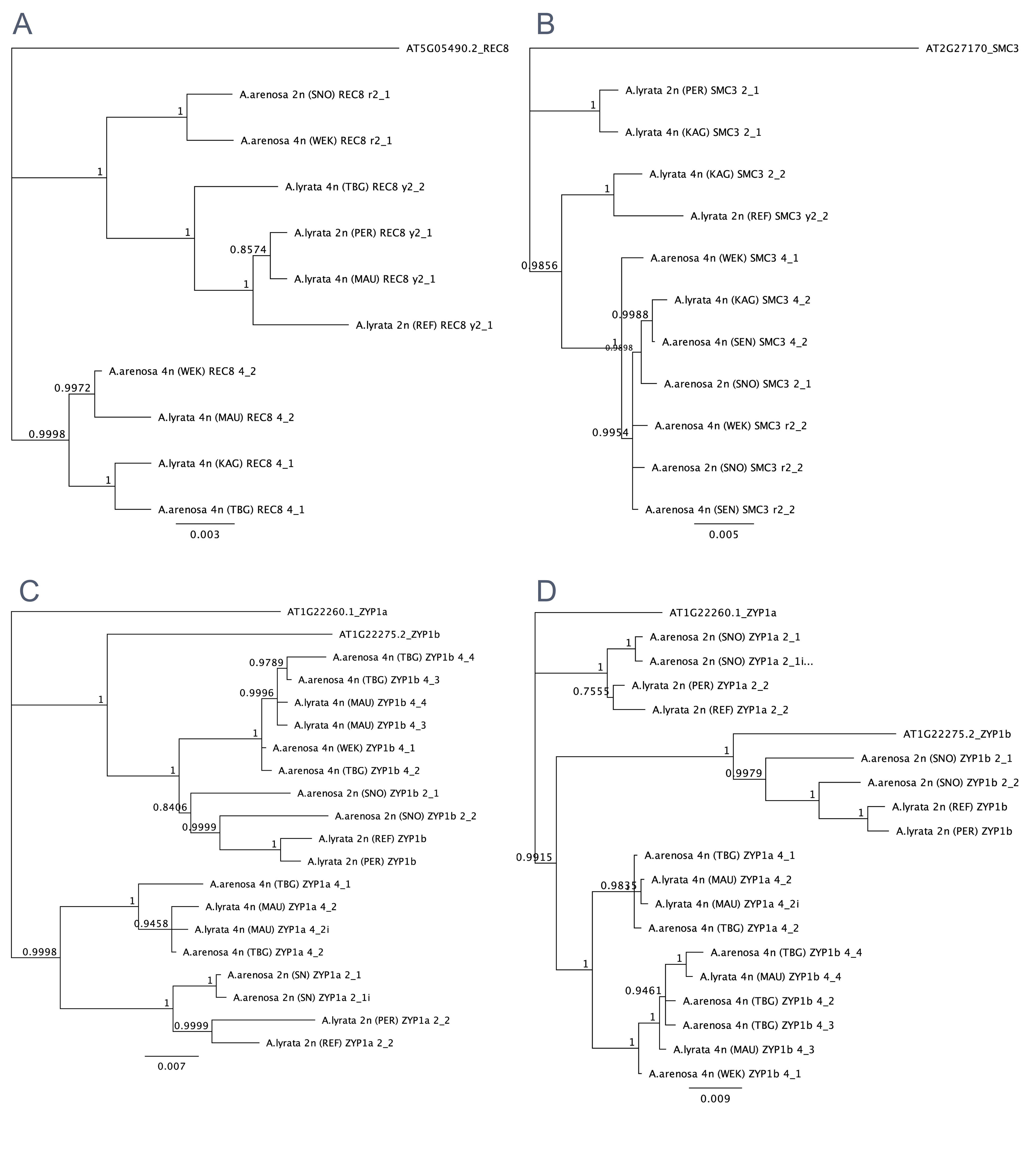

Supplement: S3 Fig — (A) REC8, (B) SMC3, (C) ZYP1a/ZYP1b 5’ end, (D) ZYP1a/ZYP1b 3’ end. Bayesian posterior probabilities are indicated at the internodes of each branch. The dissimilarity scale showing substitutions per nucleotide is located at the bottom of each tree. Diploid and tetraploid alleles are indicated by ‘2’ and ‘4’ respectively, while the putative origins are indicated by ‘r’ (A. arenosa) and ‘y’ (A. lyrata). (TIF) [file pgen.1008900.s003.tif]

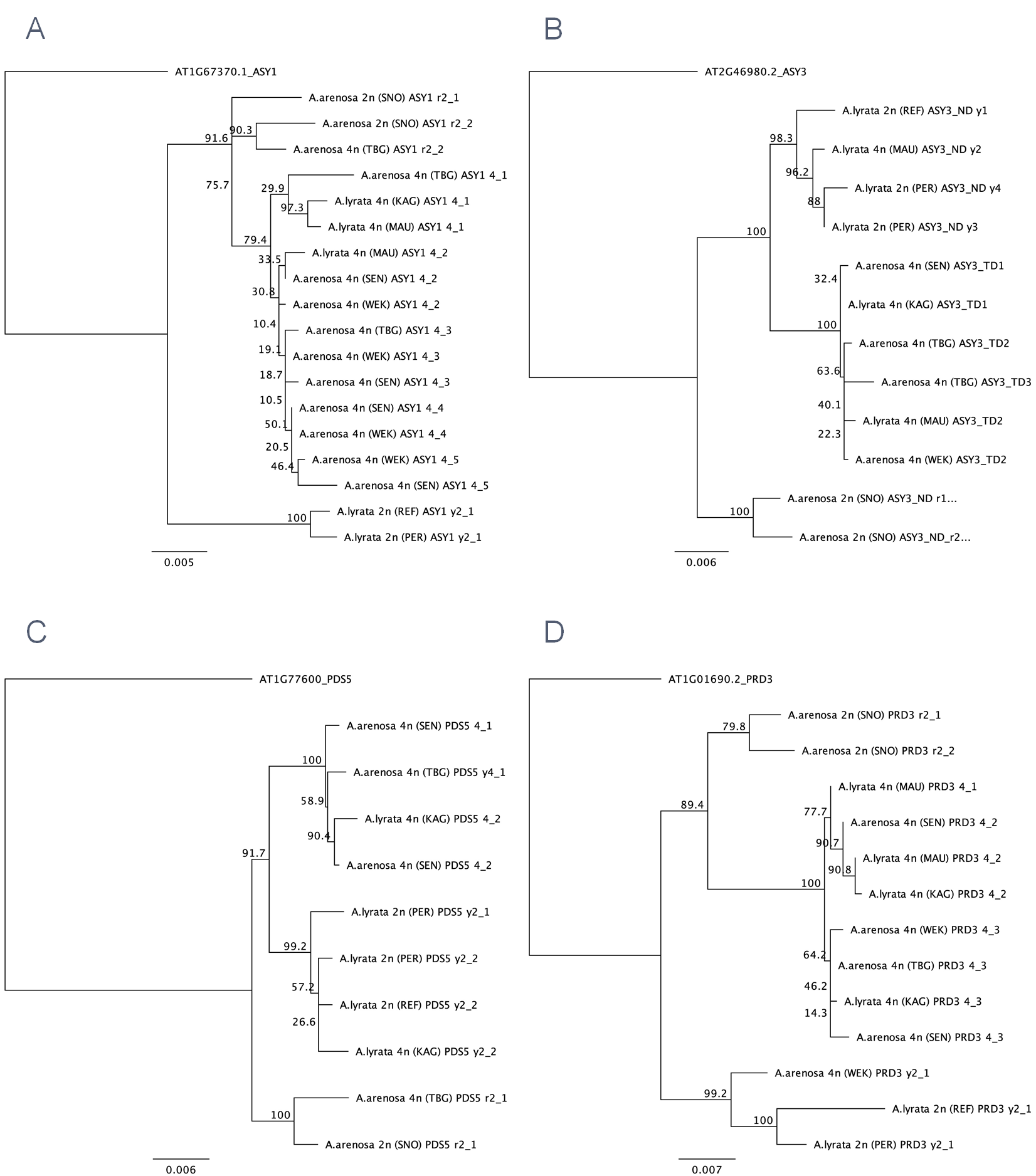

Supplement: S4 Fig — (A) ASY1, (B) ASY3, (C) PDS5b, (D) PRD3. Maximum likelihood bootstrap values are indicated at the internodes of each branch (1000 replicates). The dissimilarity scale showing substitutions per nucleotide is located at the bottom of each tree. Diploid and tetraploid alleles are indicated by ‘2’ and ‘4’ respectively (for ASY3 diploid = ND, tetraploid = TD), while the putative origins are indicated by ‘r’ (A. arenosa) and ‘y’ (A. lyrata). (TIF) [file pgen.1008900.s004.tif]

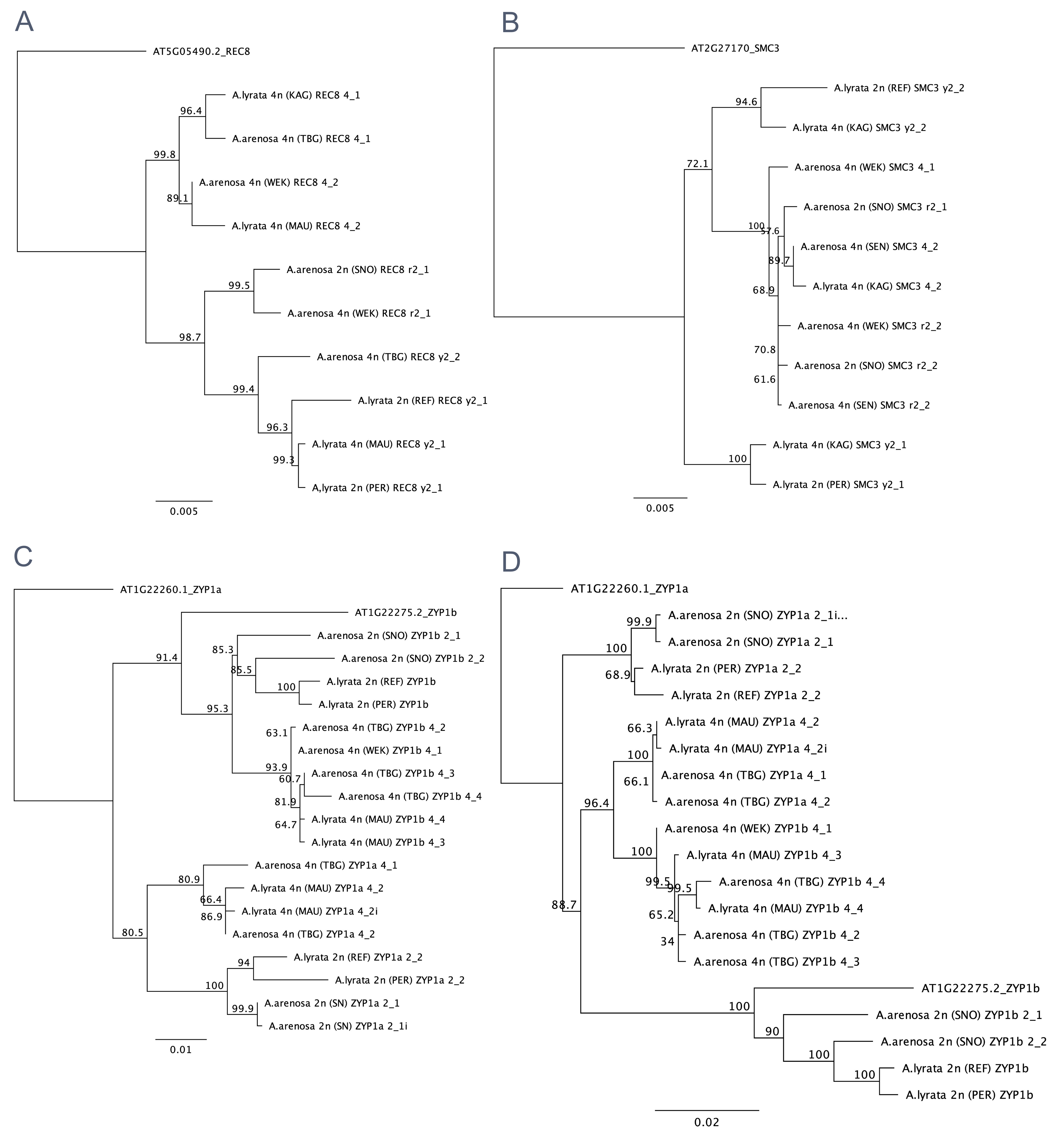

Supplement: S5 Fig — (A) REC8, (B) SMC3, (C) ZYP1a/ZYP1b 5’ end, (D) ZYP1a/ZYP1b 3’ end. Maximum likelihood bootstrap values are indicated at the internodes of each branch (1000 replicates). The dissimilarity scale showing substitutions per nucleotide is located at the bottom of each tree. Diploid and tetraploid alleles are indicated by ‘2’ and ‘4’ respectively, while the putative origins are indicated by ‘r’ (A. arenosa) and ‘y’ (A. lyrata). (TIF) [file pgen.1008900.s005.tif]

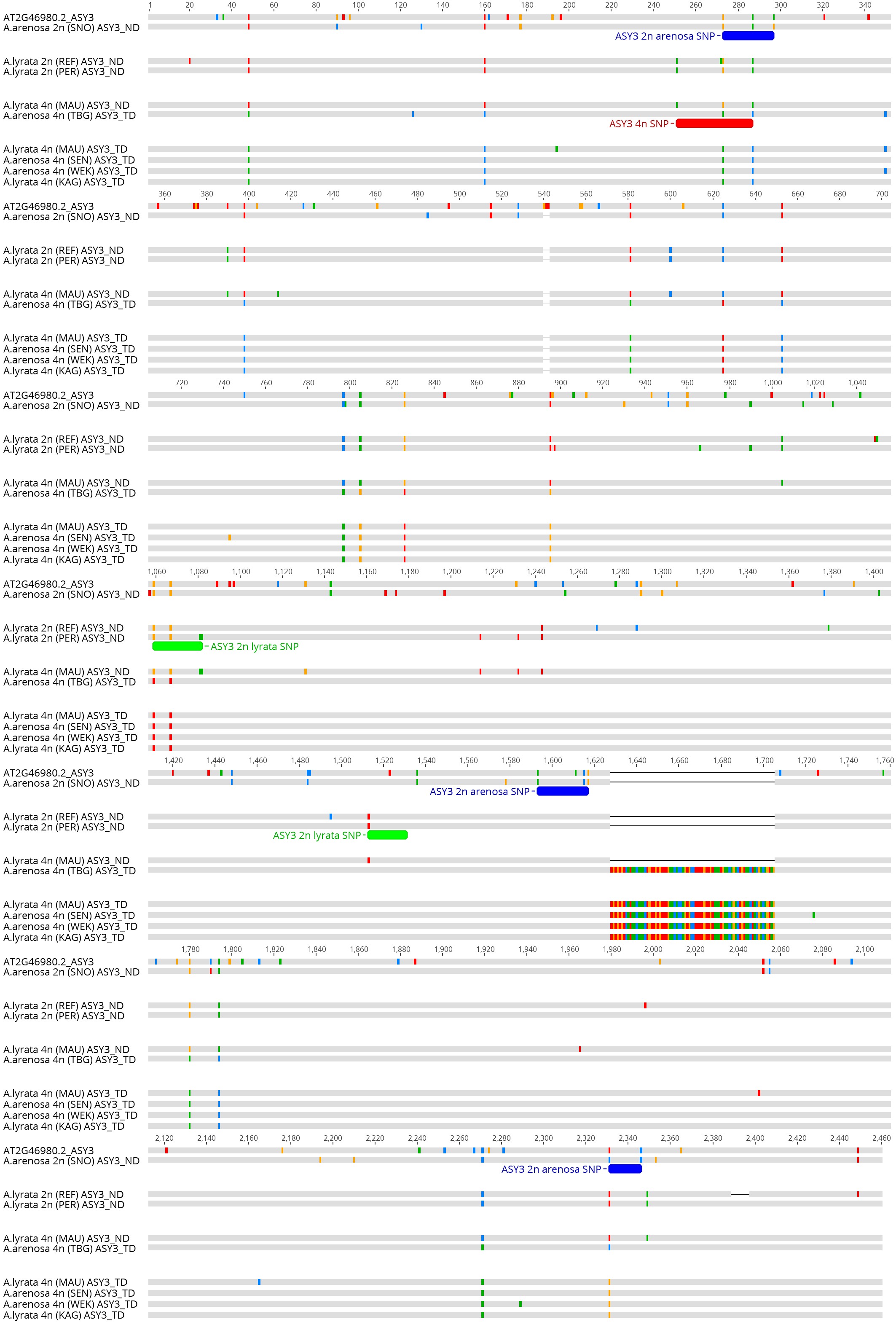

Supplement: S6 Fig — The location of diploid and autotetraploid allele specific SNPs are indicated. Coloured bars in each sequence represent base specific SNPs relative to the consensus sequence (Green = A, Blue = C, Black = G, Red = T). (JPG) [file pgen.1008900.s006.jpg]

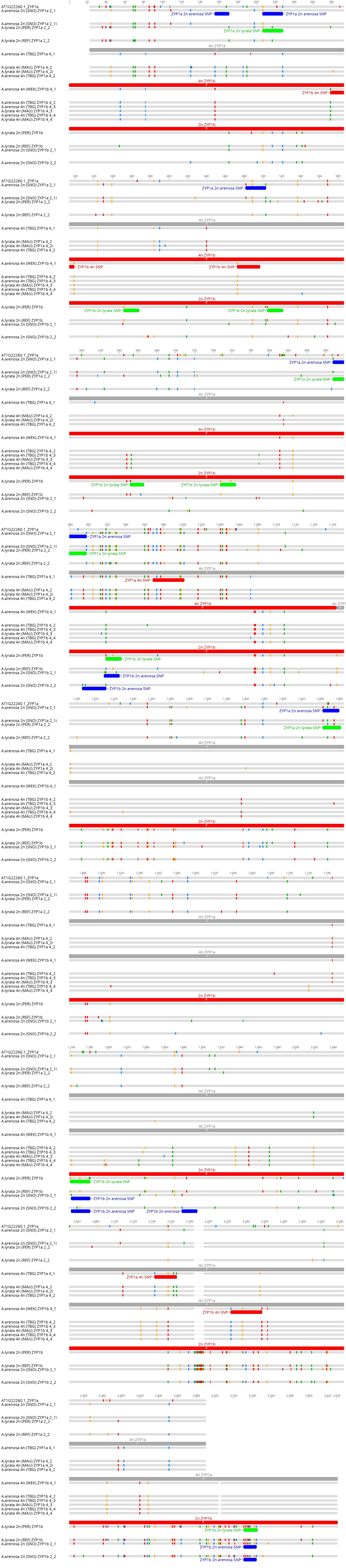

Supplement: S7 Fig — ZYP1 gene conversion (or CO) between ZYP1a (grey) and ZYP1b (red) in autotetraploid ZYP1b alleles. The location of diploid and autotetraploid allele specific SNPs are indicated. Coloured bars in each sequence represent base specific SNPs relative to the consensus sequence (Green = A, Blue = C, Black = G, Red = T). (JPG) [file pgen.1008900.s007.jpg]

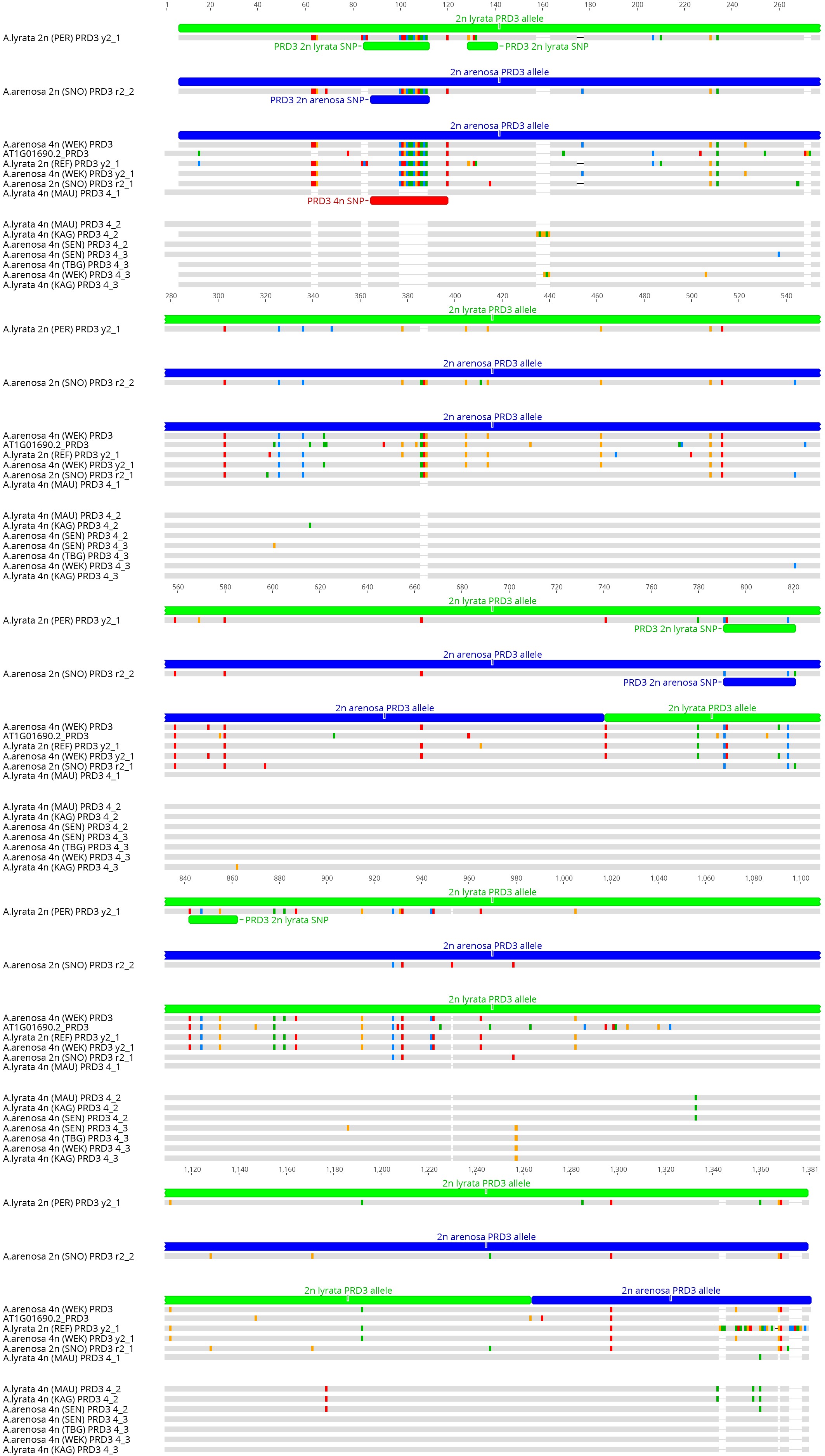

Supplement: S8 Fig — Gene conversion between PRD3 diploid A. lyrata (green) and A. arenosa (green) in tetraploid A. arenosa. The location of diploid and autotetraploid allele specific SNPs are indicated. Coloured bars in each sequence represent base specific SNPs relative to the consensus sequence (Green = A, Blue = C, Black = G, Red = T). (JPG) [file pgen.1008900.s008.jpg]

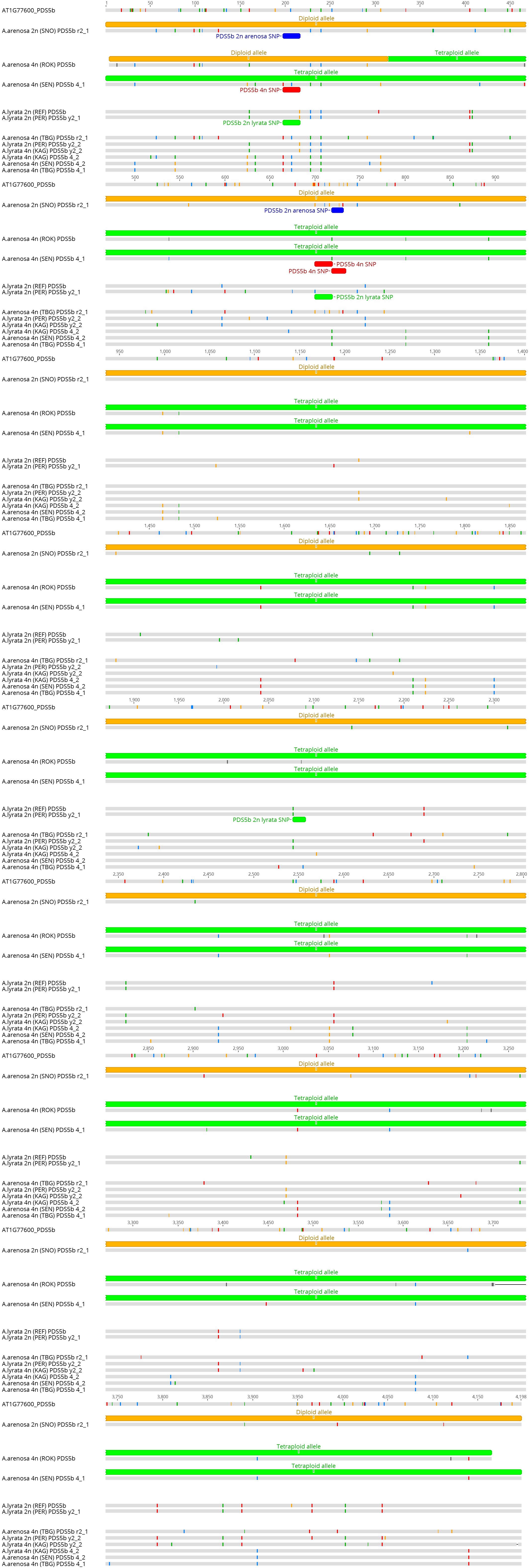

Supplement: S9 Fig — Gene conversion (or CO) between PDS5b diploid (yellow) and autotetraploid (green) alleles. The location of diploid and autotetraploid allele specific SNPs are indicated. The location of diploid and autotetraploid allele specific SNPs are indicated. Coloured bars in each sequence represent base specific SNPs relative to the consensus sequence (Green = A, Blue = C, Black = G, Red = T). (JPG) [file pgen.1008900.s009.jpg]

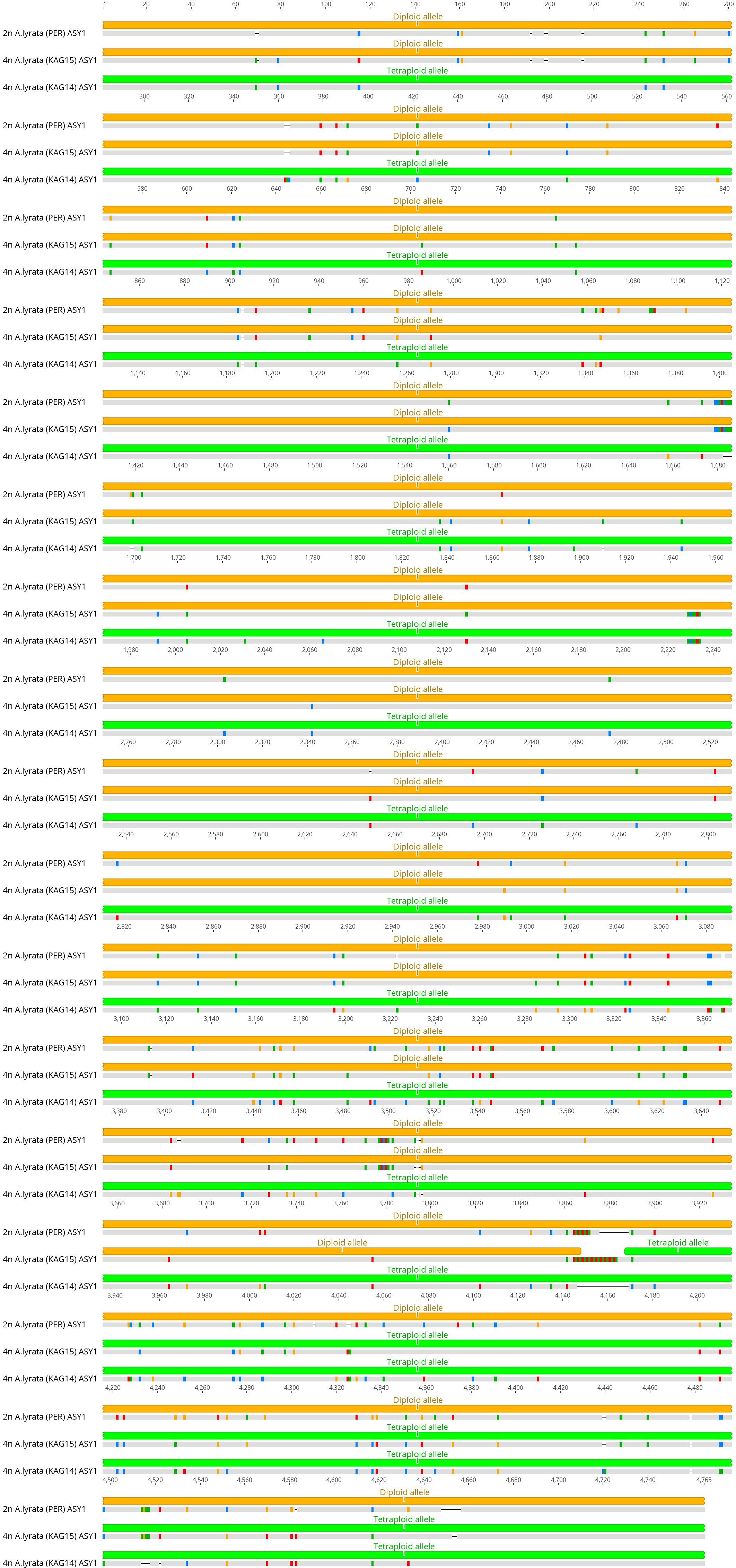

Supplement: S10 Fig — Gene conversion (or CO) between ASY1 diploid A. lyrata (yellow) and autotetraploid A. arenosa (green). Coloured bars in each sequence represent base specific SNPs relative to the consensus sequence (Green = A, Blue = C, Black = G, Red = T). (JPG) [file pgen.1008900.s010.jpg]

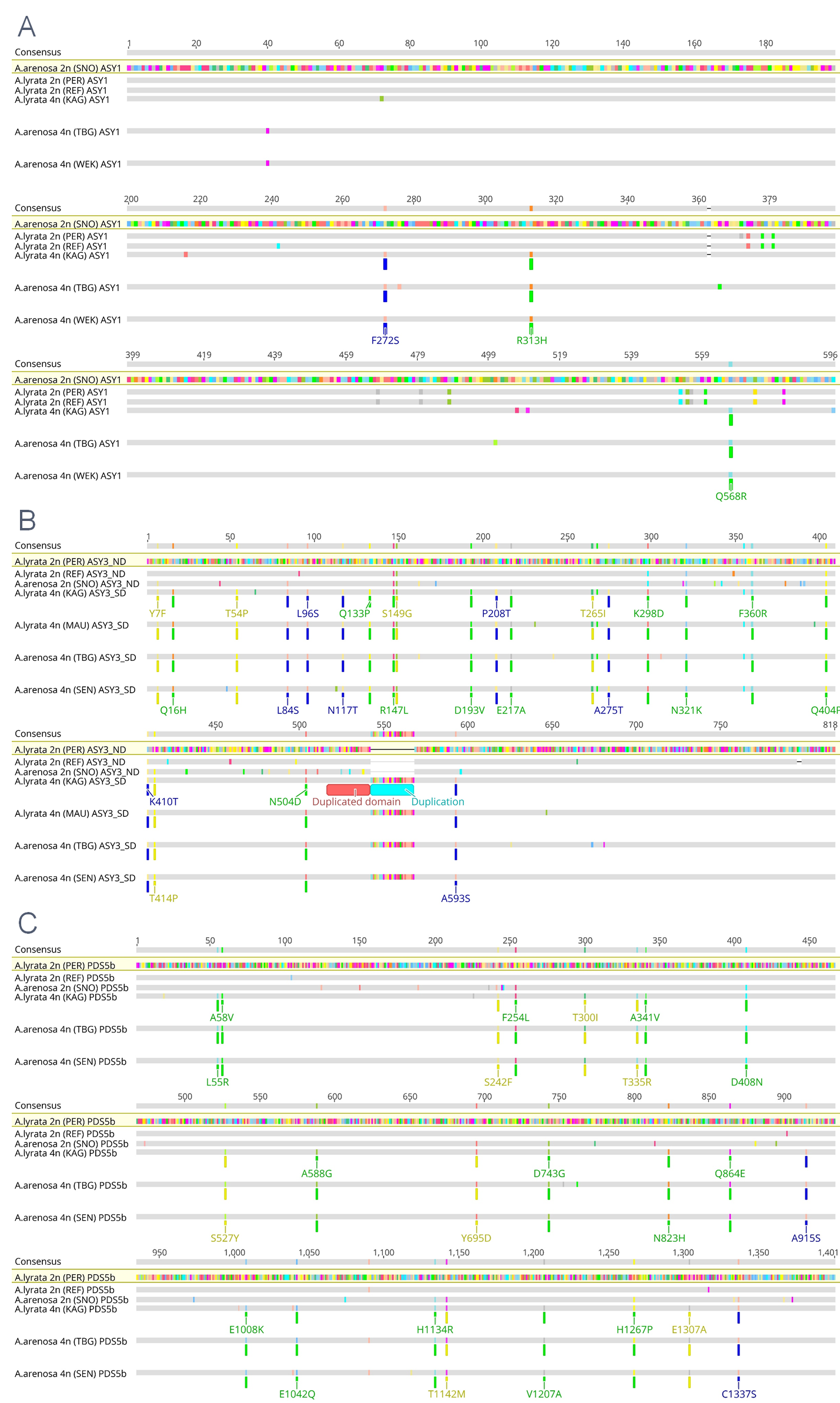

Supplement: S11 Fig — ASY1 (A), ASY3 (B), PDS5b (C), showing conserved amino acid polymorphisms in autotetraploids compared to ancestral diploid alleles. Gains, losses and no change of predicted phosphorylation sites are indicated in blue, yellow and green respectively. (TIF) [file pgen.1008900.s011.tif]

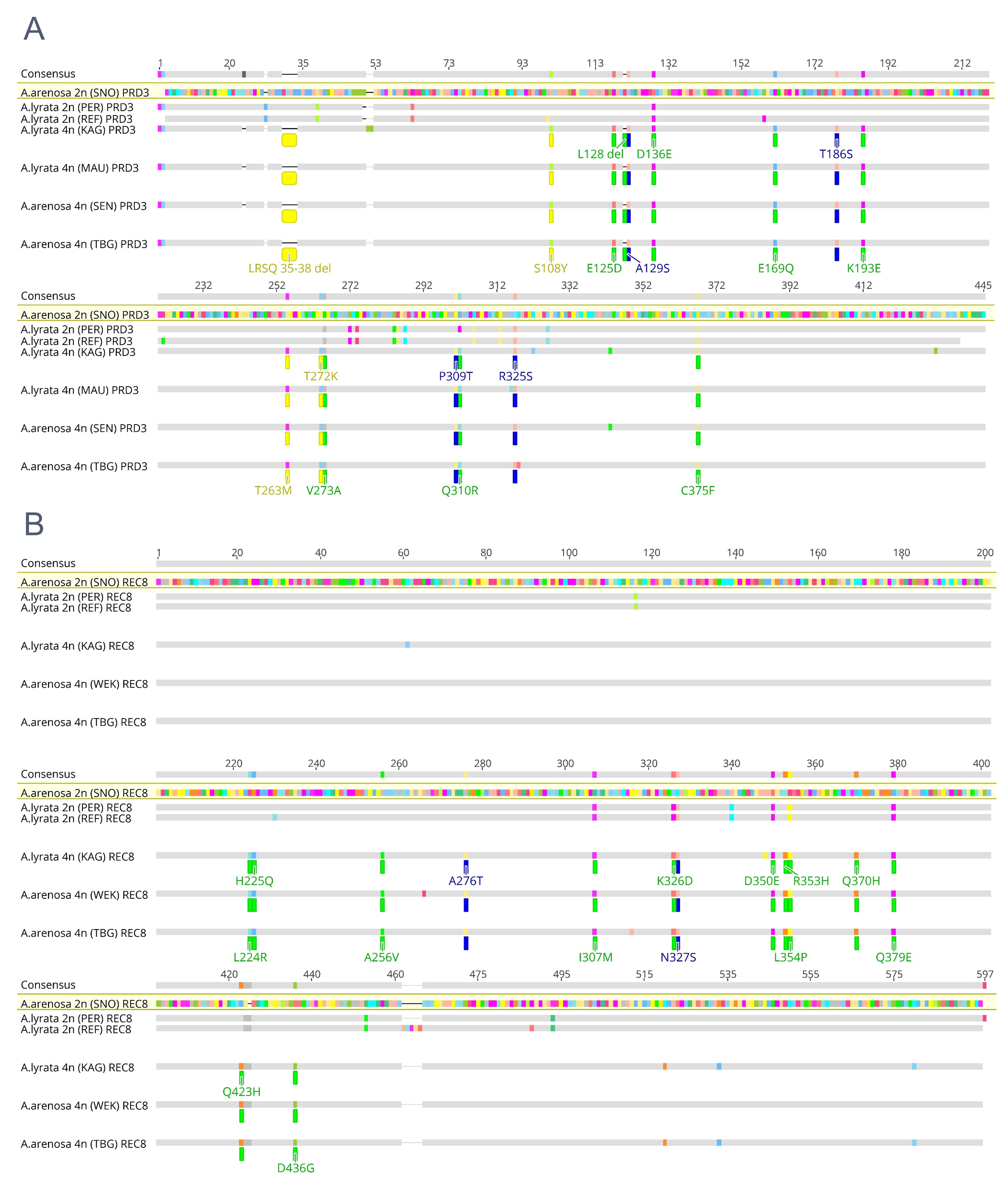

Supplement: S12 Fig — PRD3 (A), REC8 (B), showing conserved amino acid polymorphisms in autotetraploids compared to ancestral diploid alleles. Gains, losses and no change of predicted phosphorylation sites are indicated in blue, yellow and green respectively. (TIF) [file pgen.1008900.s012.tif]

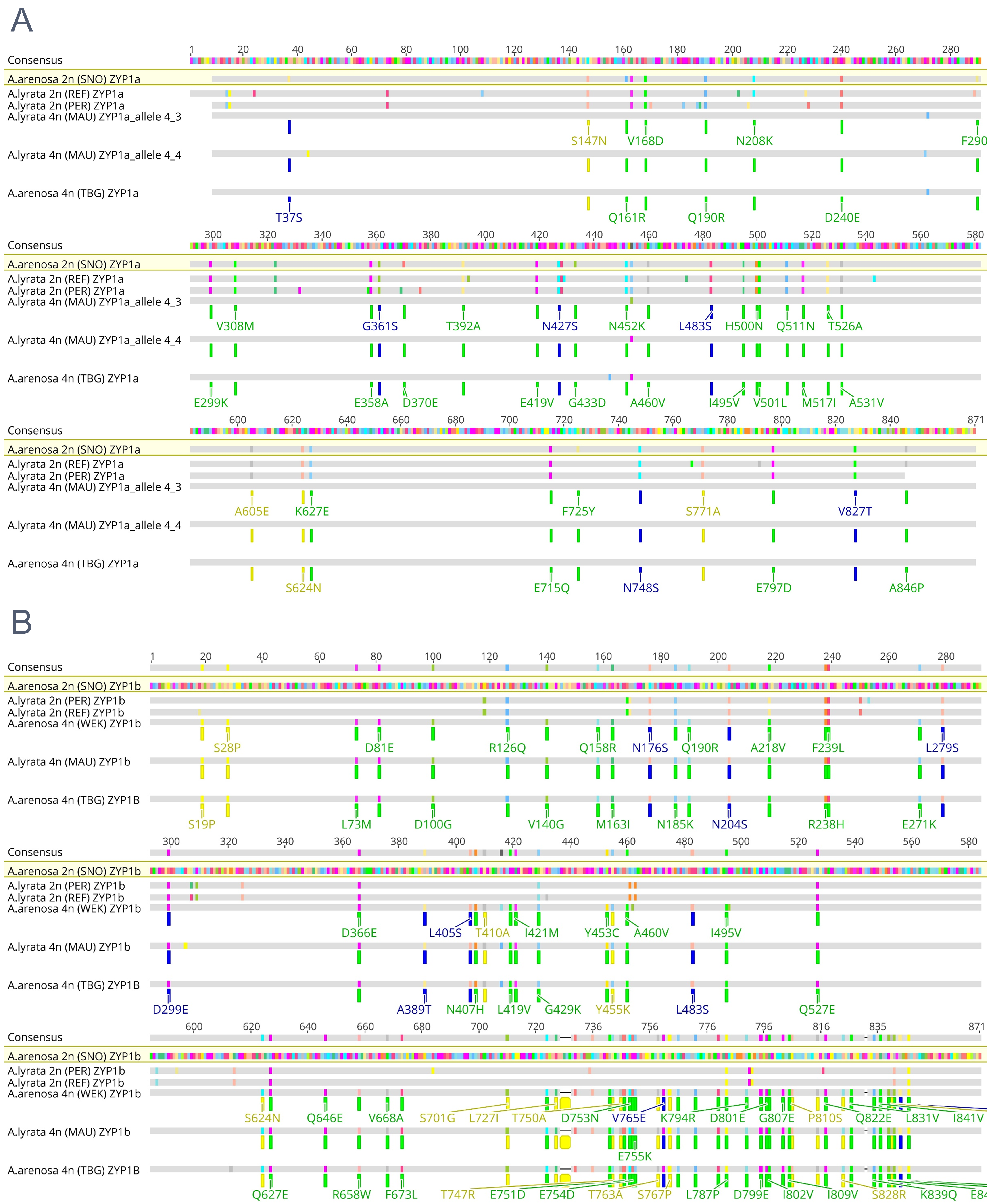

Supplement: S13 Fig — ZYP1a (A) and ZYP1b (B), showing conserved amino acid polymorphisms in autotetraploids compared to ancestral diploid alleles. Gains, losses and no change of predicted phosphorylation sites are indicated in blue, yellow and green respectively. (TIF) [file pgen.1008900.s013.tif]

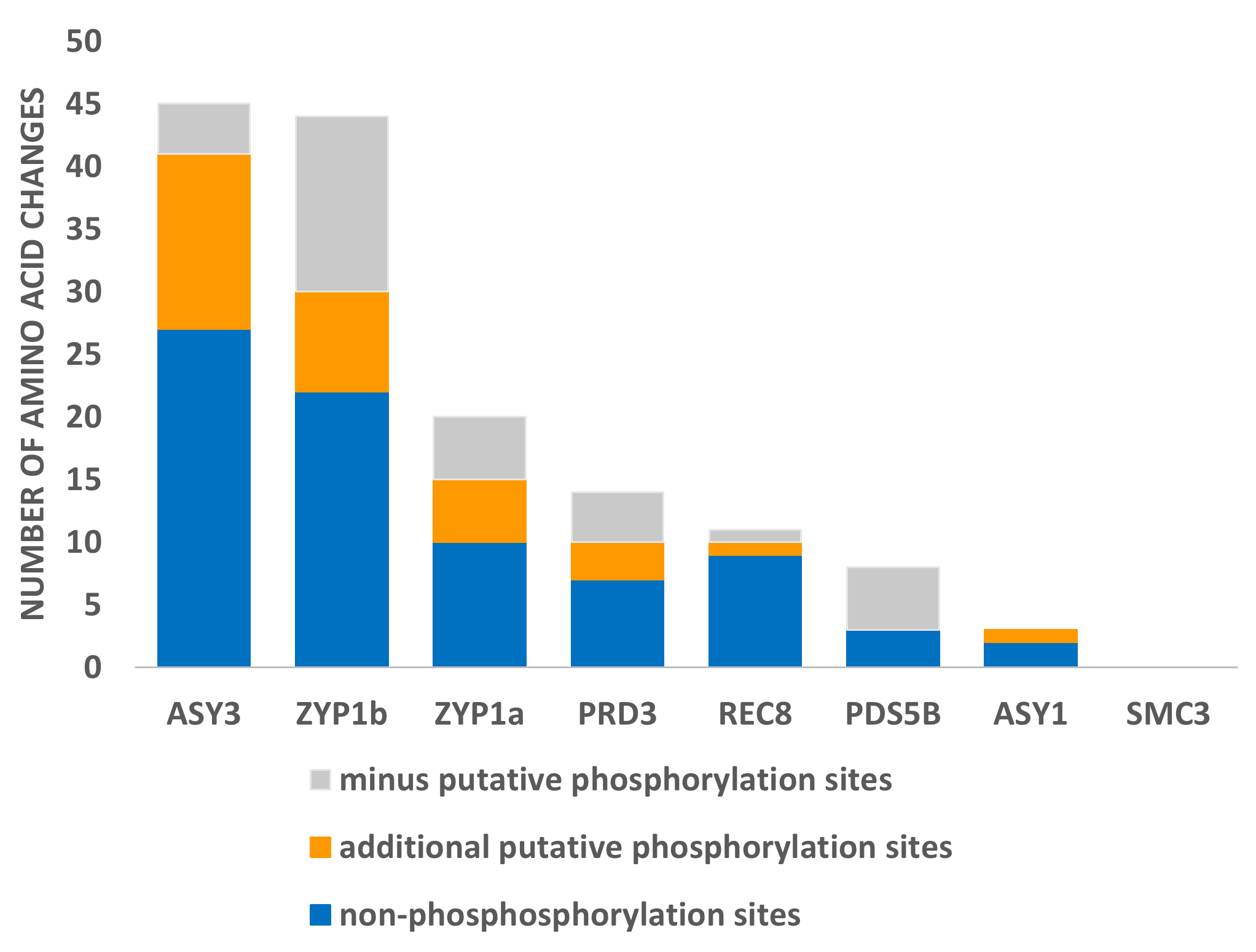

Supplement: S14 Fig — The analysis includes gains and losses of predicted serine/threonine phosphorylation sites by KinasePhos2.0 and NetPhos3.1. (TIF) [file pgen.1008900.s014.tif]
