# Supplementary material for: A novel allele of ASY3 is associated with greater meiotic stability in autotetraploid Arabidopsis lyrata
Source: PLoS Genet. 2020 Jul 15;16(7):e1008900. doi: 10.1371/journal.pgen.1008900 (PMC7392332; doi:10.1371/journal.pgen.1008900)

**S1 Table**. **Genotype and phenotype data.** Diploid lyrata alleles = ly; diploid arenosa alleles = ar and ar/ly = diploid arenosa to diploid lyrata putative gene conversions.


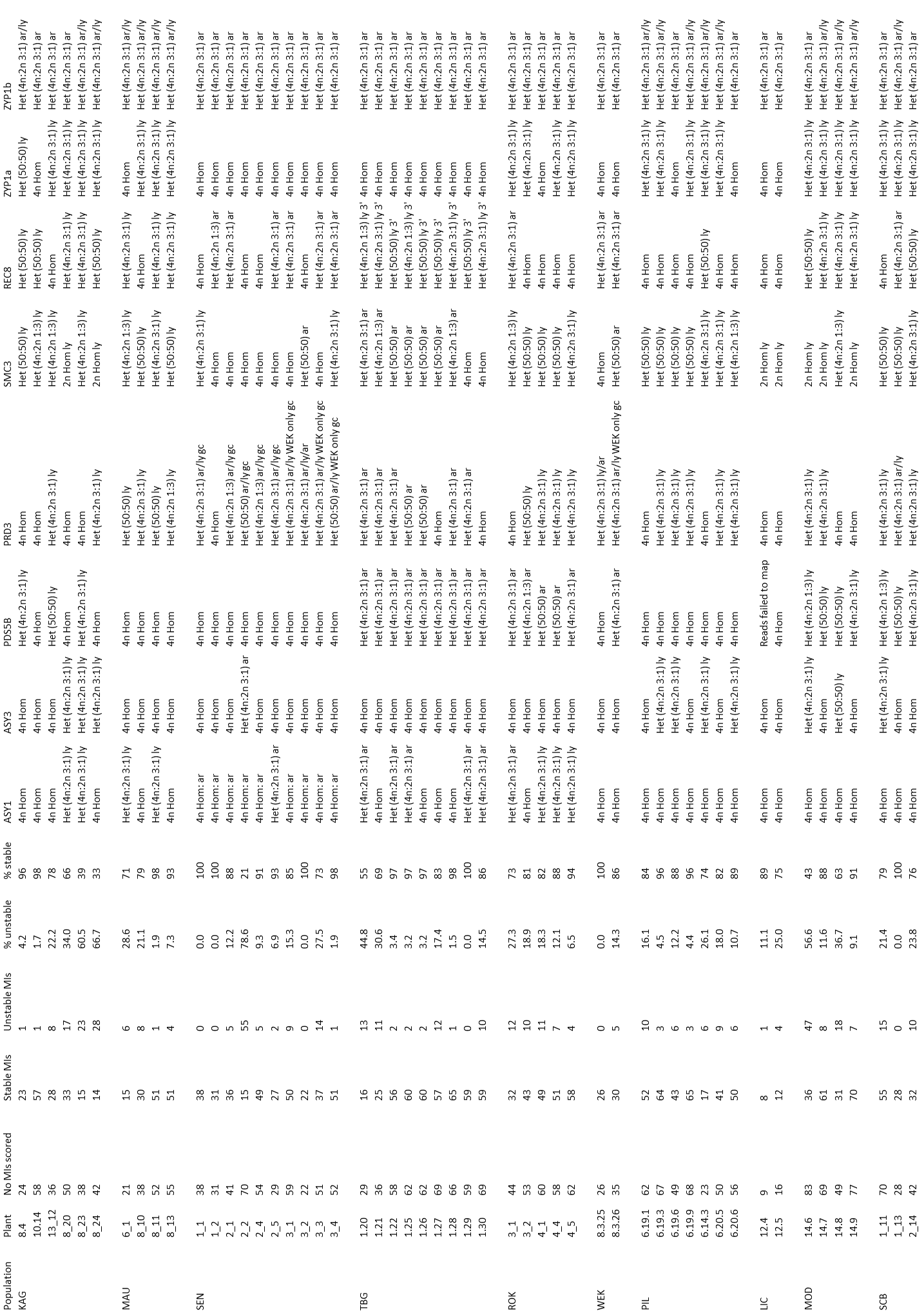

Supplement: S1 Table — Diploid lyrata alleles = ly; diploid arenosa alleles = ar and ar/ly = diploid arenosa to diploid lyrata putative gene conversions. (DOCX) [file pgen.1008900.s015.docx]
