# Supplementary material for: A novel allele of ASY3 is associated with greater meiotic stability in autotetraploid Arabidopsis lyrata
Source: PLoS Genet. 2020 Jul 15;16(7):e1008900. doi: 10.1371/journal.pgen.1008900 (PMC7392332; doi:10.1371/journal.pgen.1008900)
Supplement: S2 Table — Putative addition of serine/threonine phosphosites are highlighted in blue and loss of phosphorylation sites highlighted in yellow. (DOCX) [file pgen.1008900.s016.docx]

**S2 Table.** **Amino acid substitutions conserved in all tetraploids tested relative to diploid *A. lyrata* (PER).** Putative addition of serine/threonine phosphosites are highlighted in blue and loss of phosphorylation sites highlighted in yellow.

|  | **Substitution** | | **Property change** | **Phospho change (NetPhos 3.1)** | **Phospho change (KinasePhos 2.0)** | **Domain** |
| --- | --- | --- | --- | --- | --- | --- |
| **ASY3** | Q16H | | Polar to basic | DNA-PK to PKC | No |  |
|  | T54P | | Polar to unique | No | CKII/PDK deletion |  |
|  | L84S | | Aliphatic to polar | CDK5/p38MAPK addition | CKI addition |  |
|  | L96S | | Aliphatic to polar | PKA addition | ATM addition |  |
|  | N117T | | Conserved | PKC addition | CKII/PDK addition |  |
|  | Q133P | | Polar to unique | No | No |  |
|  | R147L | | Basic to aliphatic | No | No |  |
|  | S149G | | Polar to unique | PKA deletion | GRK deletion |  |
|  | D193V | | Acidic to aliphatic | No | No |  |
|  | P208T | | Unique to polar | No | GRK addition |  |
|  | E217A | | Acidic to aliphatic | No | No |  |
|  | T265I | | Polar to aliphatic | PKA deletion | GRK deletion |  |
|  | A275T | | Aliphatic to polar | PKC/PKG addition | GRK addition |  |
|  | K298D | | Basic to acidic | No | No |  |
|  | N321K | | Polar to basic | No | No |  |
|  | F360R | | Aromatic to basic | No | No |  |
|  | Q404P | | Polar to unique | No | No |  |
|  | K410T | | Basic to polar | No | GRK addition |  |
|  | T414P | | Polar to basic | No | CDK/PKC deletion |  |
|  | +543R | | Basic | No | No |  |
|  | +544D | | Acidic | No | No |  |
|  | +545Y | | Polar | SRC addition | FGFR1 addition |  |
|  | +546S | | Polar | PKC addition | ATM addition |  |
|  | +547F | | Non-polar | No | No |  |
|  | +548R | | Basic | No | No |  |
|  | +549R | | Basic | No | No |  |
|  | +550E | | Acidic | No | No |  |
|  | +551S | | Polar | PKA addition | ATM addition |  |
|  | +552S | | Polar | GSK3 addition | ATM addition |  |
|  | +553P | | Non-polar | No | No |  |
|  | +554E | | Acidic | No | No |  |
|  | +555P | | Non-polar | No | No |  |
|  | +556K | | Basic | No | No |  |
|  | +557E | | Acidic | No | No |  |
|  | +558D | | Acidic | No | No |  |
|  | +559L | | Non-polar | No | No |  |
|  | +560V | | Non-polar | No | No |  |
|  | +561L | | Non-polar | No | No |  |
|  | +562S | | Polar | CKII addition | PLK1 addition |  |
|  | +563D | | Acidic | No | No |  |
|  | +564S | | Polar | CKII addition | ATM addition |  |
|  | +565S | | Polar | CKII addition | CKII addition |  |
|  | +566S | | Polar | CKII addition | ATM addition |  |
|  | +567D | | Acidic | No | No |  |
|  | +568E | | Acidic | No | No |  |
|  | | L55R | Aliphatic to basic | No | No |  |

| PDS5b |  |  |  |  |  |
| --- | --- | --- | --- | --- | --- |
|  | A58V | Conserved | No | No |  |
|  | S242F | Polar to aromatic | PKC/CK1 deletion | ATM deletion | Putative peptide binding |
|  | T300I | Polar to aliphatic | No | GRK deletion | Putative peptide binding |
|  | T335R | Polar to basic | No | CK2/PDK deletion | Putative peptide binding |
|  | A341V | Conserved | No | No | Putative peptide binding |
|  | S527Y | Polar to aromatic | DNA-PK deletion | ATM deletion |  |
|  | A588G | Aliphatic to unique | No | No |  |
|  | V1207A | Conserved | No | No |  |
|  | E1304A | Acidic to aliphatic | CKII deletion | No |  |

Amino acid substitutions conserved in all tetraploids tested relative to diploid *A. lyrata* (PER). Putative addition of phosphorylation motifs are highlighted in blue and loss of phosphorylation motifs highlighted in yellow.
