# Supplementary material for: A novel allele of ASY3 is associated with greater meiotic stability in autotetraploid Arabidopsis lyrata
Source: PLoS Genet. 2020 Jul 15;16(7):e1008900. doi: 10.1371/journal.pgen.1008900 (PMC7392332; doi:10.1371/journal.pgen.1008900)
Supplement: S3 Table — Putative addition of serine/threonine phosphorylation sites are highlighted in blue and loss of phosphorylation sites highlighted in yellow. (DOCX) [file pgen.1008900.s017.docx]

**S3 Table. Amino acid substitutions in ASY3 of 2n *A. arenosa* (SNO) relative to 2n *A. lyrata* (PER).** Putative addition of serine/threonine phosphorylation sites are highlighted in blue and loss of phosphorylation sites highlighted in yellow.

|  | **ASY3** |  |  |  |  |
| --- | --- | --- | --- | --- | --- |
|  | \| Substitution \| Property change \| Phospho change (NetPhos 3.1) \| Phospho change (KinasePhos2.0) \| Domain \| \| --- \| --- \| --- \| --- \| --- \| \| L84S \| Aliphatic to polar \| CDK5/p38MAPK addition \| CK1 addition \|  \| \| R147L \| Basic to aliphatic \| No \| No \|  \| \| S149G \| Polar to unique \| PKA deletion \| GRK deletion \|  \| \| R162T \| Basic to polar \| No \| PLK1 addition \|  \| \| L172Q \| Aliphatic to polar \| No \| No \|  \| \| K298N \| Basic to polar \| CKII addition \| No \|  \| \| N321K \| Polar to basic \| No \| No \|  \| \| K342N \| Basic to polar \| No \| No \|  \| \| A352T \| Aliphatic to polar \| PKC addition \| PKA addition \|  \| \| F360R \| Aromatic to basic \| No \| No \|  \| \| Q380H \| Polar to basic \| ATM to PKC \| No \|  \| \| Q404P \| Polar to unique \| No \| No \|  \| \| K410T \| Basic to polar \| No \| GRK addition \|  \| \| T414P \| Polar to unique \| No \| PKC/CDK deletion \|  \| \| A467V \| Conserved \| No \| No \|  \| \| T478I \| Polar to aliphatic \| CKII deletion \| PKC/CDK deletion \|  \| \| M482T \| Aliphatic to polar \| No \| GRK addition \|  \| \| N494T \| Conserved \| DNA-PK deletion \| GRK addition \|  \| \| S525R \| Polar to basic \| PKA to p38MAPK \| ATM deletion \|  \| \| K530N \| Basic to polar \| No \| No \|  \| \| S538P \| Polar to unique \| CKII deletion \| ATM deletion \|  \| |  |  |  |  |
|  |  |  |  |  |  |
|  |  |  |  |  |  |
|  |  |  |  |  |  |
|  | Amino acid substitutions in ASY3 of 2n *A. arenosa* (SNO) relative to 2n *A. lyrata* (PER). Putative addition of phosphorylation motifs are highlighted in blue and loss of phosphorylation motifs highlighted in yellow. |  |  |  |  |
|  |  |  |  |  |  |
|  |  |  |  |  |  |
