# Supplementary material for: A novel allele of ASY3 is associated with greater meiotic stability in autotetraploid Arabidopsis lyrata
Source: PLoS Genet. 2020 Jul 15;16(7):e1008900. doi: 10.1371/journal.pgen.1008900 (PMC7392332; doi:10.1371/journal.pgen.1008900)
Supplement: S4 Table — Putative addition of serine/threonine phosphorylation sites are highlighted in blue and loss of phosphorylation sites highlighted in yellow. (DOCX) [file pgen.1008900.s018.docx]

**S4 Table.** **Amino acid substitutions conserved in all tetraploids tested relative to diploid *A. arenosa* (SNO).** Putative addition of serine/threonine phosphorylation sites are highlighted in blue and loss of phosphorylation sites highlighted in yellow.

| Locus | Substitution | Property change | Phospho change (NetPhos 3.1) | Phospho change (KinasePhos2.0) | Domain |
| --- | --- | --- | --- | --- | --- |
| ASY1 | F273S | Aromatic to polar | No | Addition of ATM | PHD finger |
|  | R314H | Conserved | No | Increased probability of ATM in 4n | PHD finger |
|  | Q567R | Polar to basic | DNA-PK deletion | Decreased probability of ATM in 4n |  |
| PRD3 | L35 deletion | Aliphatic deletion | No | No |  |
|  | R36 deletion | Basic deletion | No | No |  |
|  | S37 deletion | Polar deletion | DNA-PK deletion | ATM deletion |  |
|  | Q38 deletion | Polar deletion | No | No |  |
|  | S108Y | Polar to aromatic | CDC2 deletion | ATM deletion |  |
|  | L128 deletion | Aliphatic deletion | No | No |  |
|  | A129S | Aliphatic to polar | ATM addition | ATM addition |  |
|  | T263M | Polar to aliphatic | p38MAPK deletion | CDC2 deletion |  |
|  | T272K | Polar to basic | PKC deletion | GRK deletion |  |
|  | V273A | Conserved | No | No |  |
|  | P309T | Unique to polar | No | GRK addition |  |
|  | Q310R | Polar to basic | No | No |  |
|  | R325S | Basic to polar | CDC2 addition | ATM addition |  |
|  | F375C | Aromatic to polar | No | No |  |
| REC8 | L224R | Aliphatic to basic | No | No |  |
|  | H225Q | Basic to polar | No | No |  |
|  | A256V | Conserved | No | No |  |
|  | A276T | Aliphatic to polar | PKC addition | GRK addition |  |
|  | S351T | Conserved | No | ATM to GRK |  |
|  | R353H | Conserved | No | No |  |
|  | Q370H | Polar to basic | No | No |  |
|  | Q423H | Polar to basic | No | No |  |
|  | D436G | Acidic to unique | No | No |  |
|  | H538Q | Basic to polar | No | No |  |
|  | N585K | Conserved | DNA-PK deletion | No |  |
| SMC3 | There are no amino acid differences between alleles. | | | |  |
| ZYP1a | S19P | Polar to unique | PKA deletion | ATM deletion |  |
|  | N23K | Polar to basic | No | No |  |
|  | M24T | Aliphatic to polar | PKC addition | GRK addition |  |
|  | S28P | Polar to unique | PKC deletion | ATM deletion |  |
|  | N29T | Conserved | No | GRK addition |  |
|  | Q31P | Polar to unique | No | No |  |
|  | V168D | Aliphatic to acidic | No | No |  |
|  | N208K | Polar to basic | No | No |  |
|  | F290V | Aromatic to aliphatic | No | No |  |
|  | G361S | Unique to polar | CKII addition | ATM |  |
|  | E419V | Acidic to aliphatic | No | No |  |
|  | N452K | Polar to basic | CKII addition | No |  |
|  | A460V | Conserved | No | No |  |
|  | L483S | Aliphatic to polar | No | ATM addition |  |
|  | Q511N | Conserved | No | No |  |
|  | T526A | Polar to aliphatic | No | GRK deletion |  |
|  | A531V | Conserved | No | No |  |
|  | A605E | Aliphatic to acidic | PKC deletion | No |  |
|  | S771A* | Polar to aliphatic | PKC deletion | ATM deletion |  |
|  | A846P | Aliphatic to unique | No | No |  |
| ZYP1b | S19P | Polar to unique | PKA deletion | ATM deletion |  |
|  | S28P | Polar to unique | PKC deletion | ATM deletion |  |
|  | D100G | Acidic to unique | No | No |  |
|  | V140G | Aliphatic to unique | No | No |  |
|  | R238H | Conserved | No | No |  |
|  | L279S | Aliphatic to polar | CKII addition | ATM addition |  |
|  | D299E | Conserved | CKI addition | No |  |
|  | A389T | Aliphatic to polar | No | GRK addition |  |
|  | L405S | Aliphatic to polar | CKII addition | ATM addition |  |
|  | T410A | Polar to aliphatic | PKC deletion | CKII/PDK deletion |  |
|  | K416Q | Basic to polar | CKII deletion | No |  |
|  | E419V | Acidic to aliphatic | No | No |  |
|  | G429K | Unique to basic | CKI deletion | CKII to GRK |  |
|  | Y453C | Aromatic to polar | No | No |  |
|  | T455K | Polar to basic | No | GRK deletion |  |
|  | A460V | Conserved | No | No |  |
|  | L483S | Aliphatic to polar | No | ATM addition |  |
|  | V668A | Conserved | No | No |  |
|  | S710G | Polar to unique | CKII deletion | ATM deletion |  |
|  | D724N | Acidic to polar | No | No |  |
|  | S729 deletion | Polar deletion | PKC deletion | ATM deletion |  |
|  | I730 deletion | Aliphatic deletion | No | No |  |
|  | K731 deletion | Basic deletion | No | No |  |
|  | V732 deletion | Aliphatic deletion | No | No |  |
|  | A746V | Conserved | No | No |  |
|  | T747R | Polar to basic | PKC deletion | PKC/CDK deletion |  |
|  | T750A | Polar to aliphatic | CKII deletion | PKC/CDK deletion |  |
|  | T763A | Polar to aliphatic | No | CKII/PDK deletion |  |
|  | V765E | Aliphatic to acidic | p38MAPK addition | No |  |
|  | S767P | Conserved | No | Aurora deletion |  |
|  | N771K | Polar to basic | No | No |  |
|  | L787P | Aliphatic to polar | No | No |  |
|  | G807E | Unique to acidic | No | No |  |
|  | P810S | Unique to polar | PKC addition | ATM addition |  |
|  | T819A | Polar to aliphatic | No | CKII/PDK deletion |  |
|  | S828R | Polar to basic | PKC to PKA | ATM deletion |  |
|  | L831V | Conserved | No | No |  |
|  | M836 addition | Aliphatic addition | p38MAPK addition | No |  |
|  | K839Q | Basic to polar | No | No |  |
|  | I841V | Conserved | No | No |  |
|  | E844G | Acidic to unique | No | No |  |
|  | T845A | Polar to aliphatic | No | GRK deletion |  |
|  | A846G | Aliphatic to unique | No | No |  |
|  | A848T | Aliphatic to polar | PKC addition | GRK addition |  |
|  | A851P | Aliphatic to polar | No | No |  |

Amino acid substitutions conserved in all tetraploids tested relative to diploid *A. arenosa* (SNO). Putative addition of phosphorylation motifs are highlighted in blue and loss of phosphorylation motifs highlighted in yellow.

*This aa polymorphism is also in the ZYP1a C terminus of the gene converted ZYP1b
