# Supplementary material for: A novel allele of ASY3 is associated with greater meiotic stability in autotetraploid Arabidopsis lyrata
Source: PLoS Genet. 2020 Jul 15;16(7):e1008900. doi: 10.1371/journal.pgen.1008900 (PMC7392332; doi:10.1371/journal.pgen.1008900)
Supplement: S5 Table — (DOCX) [file pgen.1008900.s019.docx]

**S5 Table. Primers used for cloning and sequencing.**

| **Primer name** | **5’-3’ sequence** | **Annealing temp (°C)** | **Extension time (mins)** | **Purpose in study** |
| --- | --- | --- | --- | --- |
| ASY1_151F | AGCTGAAGGAAGCAGAGATCACTGAG | 65 | 2 | *ASY1* cDNA cloning |
| ASY1_TBG_R1 | TCAATTAGCTTGAGATTTCTGACGCTTCG | 65 | 2 | *ASY1* cDNA cloning |
| ASY1_Ref_R1 | TCAATTAGCCTGAGATTTCTGACGCTTG | 65 | 2 | *ASY1* cDNA cloning (2n lyrata only) |
| ASY3 coding_TBG_F1 | ATGAGCGACTATAGAAGCTTCGGC | 60 | 2.5 | *ASY3* cDNA cloning  (4n and 2n arenosa) |
| ASY3 coding_TBG_R1 | TCAATCATCCCGCAAACATTCTGCGAC | 60 | 2.5 | *ASY3* cDNA cloning  (4n only) |
| ASY3 coding_Ref_F1 | ATGAGCGACTATAGAAGCTACGGC | 60 | 2.5 | *ASY3* cDNA cloning  (2n lyrata only) |
| ASY3 coding_Ref_R1 | TCAATCATCCCTCAAACATTCTGCGAC | 60 | 2.5 | *ASY3* cDNA cloning  (2n only) |
| PDS5 coding_152F | GAAAACTCCGACGCAGATTGTTTCCG | 60 | 4.5 | *PDS5b* cDNA cloning |
| PDS5 coding_Ref_R1 | GTTCTCTGTCTACGACTTCTTCTGAGTC | 60 | 4.5 | *PDS5b* cDNA cloning |
| PRD3 coding_TBG_F1 | ATGAATATAAACAAAGCCTGCGATCTG | 61 | 3.5 | *PRD3* cDNA cloning |
| PRD3 coding_R1 | CATGAGTTGTTCCAATAGTTCATTCAGAATC | 61 | 3.5 | *PRD3* cDNA cloning |
| SMC coding F2 | CGAAGGATTTAAGAGTTACAAAGAGCAAGTTG | 60 | 3.5 | *SMC3* cDNA cloning |
| SMC coding R1 | CGAGAAAGATCAGTCCCACGATACCTGA | 60 | 3.5 | *SMC3* cDNA cloning |
| SYN1 coding_F1 | GGATGGCCGCTACGTTGCAC | 60 | 2 | *REC8* cDNA cloning |
| SYN1 coding_TBG_R1 | TTACATGTTTGGTCCTCTAGCAATGAG | 60 | 2 | *REC8* cDNA cloning |
| ZYP1a coding Ref F1 | CGGCGATGAAGAGCTTAGATAAACC | 60 | 3 | *ZYP1a* cDNA cloning |
| ZYP1a coding R1 | TCAATCAAATGCATACGGATCATCAGCG | 60 | 3 | *ZYP1a* cDNA cloning |
| ZYP1b coding Ref F1 | GGCGATGAAGAGCTTGGATCAACT | 60 | 3 | *ZYP1b* cDNA cloning |
| ZYP1b coding R1 | TCAATCAAATGCATAGGGATCATCAGCA | 60 | 3 | *ZYP1b* cDNA cloning |
| ASY3_6701F | TGCCAACTTAGGTCGCAAAAGCACAG |  |  | *ASY3* cDNA sequencing |
| PDS5_925F | ACTGATCAGGTCGATGTGC |  |  | *PDS5b* cDNA sequencing |
| PDS5_2028F | AGATGCTTCCGAGGCAG |  |  | *PDS5b* cDNA sequencing |
| PDS5_3663R | CTGTACAACGATGATGGCA |  |  | *PDS5b* cDNA sequencing |
| PRD3_2459F | GACTAGCTTGAAGATGCCTGA |  |  | *PRD3* cDNA sequencing |
| PRD3_5159R | CAAGAACTAAGTCATATTCCTCAG |  |  | *PRD3* cDNA sequencing |
| SMC3_824F | CCTTAGATGAGTCCCTGAAAGAGC |  |  | *SMC3* cDNA sequencing |
| SMC3_2392R | GAACTAGGCAAACTATCATCG |  |  | *SMC3* cDNA sequencing |
| ZYP1_1124F | AGTGTTCAGTTGAGTGCAG |  |  | *ZYP1a/ZYP1b* cDNA sequencing |
| ASY1_all_671F | CGTTAATCAGCTGGAGTTGTTGC | 60 | 2.5 | *ASY1* PCR for MiSeq |
| ASY1_all_R2 | AGGTGGTTATATGGTGTCTGATAGAGG | 60 | 2.5 | *ASY1* PCR for MiSeq |
| ASY3_all_F | CGGCAGTAACTATCACCCATCAAGTC | 63 | 2.5 | *ASY3* PCR for MiSeq |
| ASY3_all_R | GCGACAATCATCTTCAGCTGCAG | 63 | 2.5 | *ASY3* PCR for MiSeq |
| PDS5_152F | GAAAACTCCGACGCAGATTGTTTCCG | 55 | 10 | *PDS5b* PCR for MiSeq |
| PDS5_all_R1 | CAATATTGTCACTTGTGTCGGTC | 55 | 10 | *PDS5b* PCR for MiSeq |
| PRD3_all_F2 | AAGCCTGCGATCTGAAATCTAT | 55 | 3 | *PRD3* PCR for MiSeq |
| PRD3_all_R2 | CCAGTTTGAGTCTATTGCATCCA | 55 | 3 | *PRD3* PCR for MiSeq |
| SMC3_all_F1 | TATTATCGAAGGATTTAAGAGTTACAAAGAGC | 58 | 4 | *SMC3* PCR for MiSeq |
| SMC3_all_R1 | GGTATCGTGGGACTGATCTTTCTCG | 58 | 4 | *SMC3* PCR for MiSeq |
| SYN1_coding_F1 | GGATGGCCGCTACGTTGCAC | 55 | 3 | *REC8* PCR for MiSeq |
| SYN1_all_R1 | GGTCCTCTAGCAATGAGAATGTC | 55 | 3 | *REC8* PCR for MiSeq |
| ZYP1_all_F1 | CCTCCGGAAGTTTCTCGAAT | 59 | 3 | *ZYP1a/ZYP1b* PCR for MiSeq |
| ZYP1_all_R1 | AATCAAATGCATAGGGATCATCAGC | 59 | 3 | *ZYP1a/ZYP1b* PCR for MiSeq |
| ASY3_DEL_2960F | GCGTCAGATACAGAAAGGAGATTGCT | 63 | 4 | *ASY3 DEL* gDNA cloning |
| ASY3_DEL_900R | ATAGCATTTAACCAGACAATACAGGAG | 63 | 4 | *ASY3 DEL* gDNA cloning |
| Exon1_SD_F2 | TTCTCAAGGGACAACAGGCCAAGCGTC | 69 | 0.5 | Partial *ASY3 DEL* cDNA cloning |
| Exon2_SD_R2 | TGGCCTAACACTGGTGAATCTTCTCCTGAACC | 69 | 0.5 | Partial *ASY3 DEL* cDNA cloning |
| ASY3_DEL_2960F | GCGTCAGATACAGAAAGGAGATTGCT | 55 | 2 | *ASY3 DEL* 3’ RACE-PCR |

Primers used for cloning and sequencing.
