# Supplementary material for: A novel allele of ASY3 is associated with greater meiotic stability in autotetraploid Arabidopsis lyrata
Source: PLoS Genet. 2020 Jul 15;16(7):e1008900. doi: 10.1371/journal.pgen.1008900 (PMC7392332; doi:10.1371/journal.pgen.1008900)
Supplement: S6 Table — (DOCX) [file pgen.1008900.s020.docx]

**Stable 6.**

| **EnsemblPlants ID** | **Gene** | **Location** |
| --- | --- | --- |
| Scaffold_201546.1 | *ASY1* | Chr2:12427364:12431683 |
| Fgenesh2_kg.4_2929 | *ASY3* | Chr4:22845898:22850613 |
| Scaffold_202722.1 | *PDS5b* | Chr2:17706052:17715900 |
| Scaffold_0001_57 | *PRD3* | Chr1:220193:225616 |
| Fgenesh1_pg.C_scaffold_4000806 | *SMC3* | Chr4:11123922:11131646 |
| Fgenesh1_pm.C_scaffold_6000400 | *REC8* | Chr6:2001440:2005979 |
| Fgenesh00000004233 | *ZYP1a* | Chr1:9779047:9784303 |
| Scaffold_102525.1 | *ZYP1b* | Chr1:9786597:9791542 |
